# Genomic Insights into the Mobilome and Resistome of Sentinel Microorganisms Originating from Farms of Two Different Swine Production Systems

**DOI:** 10.1128/spectrum.02896-22

**Published:** 2022-11-15

**Authors:** Oscar Mencía-Ares, Maria Borowiak, Héctor Argüello, José Francisco Cobo-Díaz, Burkhard Malorny, Avelino Álvarez-Ordóñez, Ana Carvajal, Carlus Deneke

**Affiliations:** a Department of Animal Health, Veterinary Faculty, Universidad de León, León, Spain; b Department of Biological Safety, German Federal Institute for Risk Assessment, Berlin, Germany; c Department of Food Hygiene and Technology, Veterinary Faculty, Universidad de León, León, Spain; d Institute of Food Science and Technology, Universidad de León, León, Spain; University of Guelph

**Keywords:** antimicrobial resistance, *Campylobacter coli*, *Escherichia coli*, *Enterococcus* spp., mobile genetic element, pigs, *Staphylococcus* spp., sustainable farming, whole-genome sequencing

## Abstract

Antimicrobial resistance (AMR) is a threat to public health due to long-term antimicrobial use (AMU), which promotes the bacterial acquisition of antimicrobial resistance determinants (ARDs). Within food-producing animals, organic and extensive Iberian swine production is based on sustainable and eco-friendly management systems, providing an excellent opportunity to evaluate how sustained differences in AMU impact the development and spread of AMR. Here, through a whole-genome sequencing approach, we provide an in-depth characterization of the resistome and mobilome and their interaction in 466 sentinel bacteria, namely, Escherichia coli, Enterococcus spp., Campylobacter coli, and Staphylococcus spp., recovered from 37 intensive and organic-extensive pig farms. Both ARDs and mobile genetic elements (MGEs) were primarily taxon-associated, with higher similarities among bacteria which were closely phylogenetically related. E. coli exhibited the most diverse resistome and mobilome, with 85.4% mobilizable ARDs, 50.3% of which were plasmid-associated. Staphylococcus spp. exhibited a broad repertoire of ARDs and MGEs, with 52.3% of its resistome being mobilizable. Although *Enterococcus* spp. carried the highest number of ARDs per isolate and its plasmidome was similar in size to that of E. coli, 43.7% of its resistome was mobilizable. A narrow spectrum of ARDs constituted the C. coli resistome, with point mutations as its main AMR driver. A constrained AMU, as observed in organic-extensive herds, determined a reduction in the quantitative composition of the resistome and the complexity of the resistome-mobilome interaction. These results demonstrate taxon-associated AMR-MGE interactions and evidence that responsible AMU can contribute to reducing AMR pressure in the food chain.

**IMPORTANCE** This study provides the first integral genomic characterization of the resistome and mobilome of sentinel microorganisms for antimicrobial resistance (AMR) surveillance from two different swine production systems. Relevant differences were observed among taxa in the resistomes and mobilomes they harbored, revealing their distinctive risk in AMR dissemination and spread. Thus, Escherichia coli and, to a lesser extent, Staphylococcus spp. constituted the main reservoirs of mobilizable antimicrobial resistance genes, which were predominantly plasmid-associated; in contrast to Campylobacter coli, whose resistome was mainly determined by point mutations. The reduced complexity of mobilome-resistome interaction in *Enterococcus* spp. suggested its limited role in AMR dissemination from swine farms. The significant differences in antimicrobial use among the studied farms allowed us to assess the suitability of whole-genome sequencing as a rapid and efficient technique for the assessment of mid- to long-term on-farm interventions for the reduction of antimicrobial use and the evaluation of AMR status.

## INTRODUCTION

The rise of antimicrobial resistance (AMR) is one of the largest threats to global health and food safety, to the point that the World Health Organization (WHO) foresees entry into the post-antimicrobial era during the current century (1). AMR development and spread occurs by the acquisition of AMR determinants (ARDs), either through the selection of mutations in bacterial genomes or the acquisition of AMR genes (ARGs) through horizontal gene transfer (HGT) events (2). The latter is of major concern because it contributes to the capture, accumulation, and dissemination of ARGs due to the frequent involvement of mobile genetic elements (MGEs), which promote and enable intracellular and intercellular ARG mobility (3, 4).

Long-term antimicrobial use (AMU), particularly its misuse, is the main driver of the emergence, enrichment, and exchange of AMR in anthropogenic environments, where bacterial genomes adapt to these challenging conditions (5, 6). Industrialized livestock herds are potential hotspots for the enrichment and exchange of ARDs (6), and recent studies using high-throughput sequencing methods support this statement, particularly in swine production (7, 8). In this regard, and taking into account current policies aimed at reducing AMU in livestock (9), non-industrialized livestock models offer an opportunity to compare and extrapolate differences in AMR burdens between producers with frequent and limited AMU and estimate the impact of different approaches on AMR reversal.

Traditional extensive Iberian pig production in Spain is defined by eco-friendly and sustainable practices, including constrained use of antimicrobials (10), which has been maintained for decades. Thus, this extensive production system offers an ideal means to study how sustained differences in AMU have impacted the microbial communities colonizing animals and the farm environment, including their genomic content and their MGE and ARD cargo. Indeed, in a previous study, we demonstrated, through whole-metagenome sequencing analyses, highly significant differences in resistome profiles between intensive and extensive Iberian pig farms, and we associated these differences with AMU and MGE abundance on farms (8). However, although metagenomics contributes to an overview of resistome diversity and abundance, this technique provides information on predominant taxa and ARDs, with limited contextualization of the ARDs detected, particularly their associations with MGEs and taxa.

Because AMR bacteria and ARDs often cross environments and species boundaries, it is critical to decipher the potential enrichment and exchange of ARDs in animal- and human-associated bacteria. AMR surveillance in targeted indicator and zoonotic bacteria (i.e., sentinel microorganisms) in food-producing animals provides critical information for assessing the potential transmission of AMR bacteria and ARDs to humans, either directly or indirectly through the food chain, soil, or water (11). Whole-genome sequencing (WGS) approaches are being gradually implemented to complement or replace traditional AMR surveillance schemes based on phenotypic antimicrobial susceptibility testing (AST) and molecular monitoring of certain critically important ARGs, such as *bla*_CTX-M_, *mecA*, or *mcr* (12) because WGS allows the characterization of an entire set of ARDs and MGEs present in a microorganism, which can provide a wealth of information on the epidemiology of AMR emergence and spread (13).

Here, using a WGS approach, we provide an in-depth characterization of the resistome and mobilome in a selection of 466 sentinel isolates belonging to four different taxa, namely, Escherichia coli, *Enterococcus* spp., Campylobacter coli, and Staphylococcus spp., recovered from 37 intensive and organic-extensive Spanish swine herds. Our results reveal overall and particular dissimilarities in AMR development due to sustained differences in AMU between both production systems and show the benefits of WGS-based AMR surveillance of combined zoonotic and indicator bacteria as sentinel microorganisms for monitoring the effect of AMU intervention strategies, with E. coli and, to a lesser extent, Staphylococcus spp., as the most relevant microorganisms due to the great diversity of their resistome and mobilome.

## RESULTS

### The microbiome of swine farms encompassed a wide diversity of isolates from the selected indicator and zoonotic bacteria.

A set of 466 indicator and zoonotic bacteria recovered from 37 Spanish swine farms from two different production systems, namely, intensive (*n *=* *18 farms) and organic-extensive (*n *=* *19), were subjected to short-read WGS. These bacteria belonged to four different taxa: *Enterococcus* spp. (*n *=* *146 isolates), Escherichia coli (*n *=* *145), Campylobacter coli (*n *=* *92), and Staphylococcus spp. (*n *=* *83). A detailed identification of sentinel microorganisms by species and, within species, by production system is shown in Table 1.

**TABLE 1:** Identification of 466 sentinel bacteria isolates belonging to four taxa from two different swine production systems

Bacterial taxon (*n *=* *466), no. of isolates	Production system	
	Intensive (*n *=* *18)	Organic-extensive (*n *=* *19)
Escherichia coli (145)	71	74
Campylobacter coli (92)	47	45
Enterococcus spp. (146)		
E. faecium (105)	46	59
E. faecalis (23)	16	7
E. hirae (9)	3	6
E. thailandicus (5)	4	1
E. durans (3)	1	2
E. mundtii (1)	1	0
Staphylococcus spp. (83)		
S. haemolyticus (25)	14	11
S. chromogenes (15)	7	8
S. hyicus (13)	4	9
S. aureus (10)	4	6
S. pasteuri (4)	3	1
S. cohnii (3)	2	1
S. devriesei (3)	0	3
S. saprophyticus (2)	1	1
S. simulans (2)	0	2
S. xylosus (2)	2	0
S. agnetis (1)	0	1
S. epidermidis (1)	0	1
S. lentus (1)	1	0
S. warneri (1)	1	0

The sequenced isolates were highly diverse, regardless of taxa, as observed by their clustering using the whole-genome average nucleotide identity (ANI) method and—when a scheme was available—by multilocus sequence typing (MLST). This phylogenetic diversity was particularly evident in E. coli and *Enterococcus* spp. (Fig. S1 and S2). In the former, 73 different sequence types (STs) were identified, of which 51 were detected only once. ST-10, the most prevalent, accounted for 9.0% of E. coli isolates. In C. coli (Fig. S3), 28 different STs were identified, with ST-854 being the most prevalent (28.3%), mainly in isolates recovered from fecal samples (*P* < 0.001). In Staphylococcus spp., we observed species-dependent diversity (Fig. S4). Thus, within the main four Staphylococcus species detected, *S. chromogenes* and *S. hyicus* isolates exhibited a wide diversity, while *S. haemolyticus* and S. aureus isolates were distributed into two main clusters, revealing higher clonality. The latter result suggests the predominance of particular STs of these two species on swine farms, as evidenced by the dominance of the livestock-associated methicillin-resistant S. aureus (MRSA) ST-398.

### The resistome is taxon-dependent, with a clear influence of the production system.

**FIG 1:**
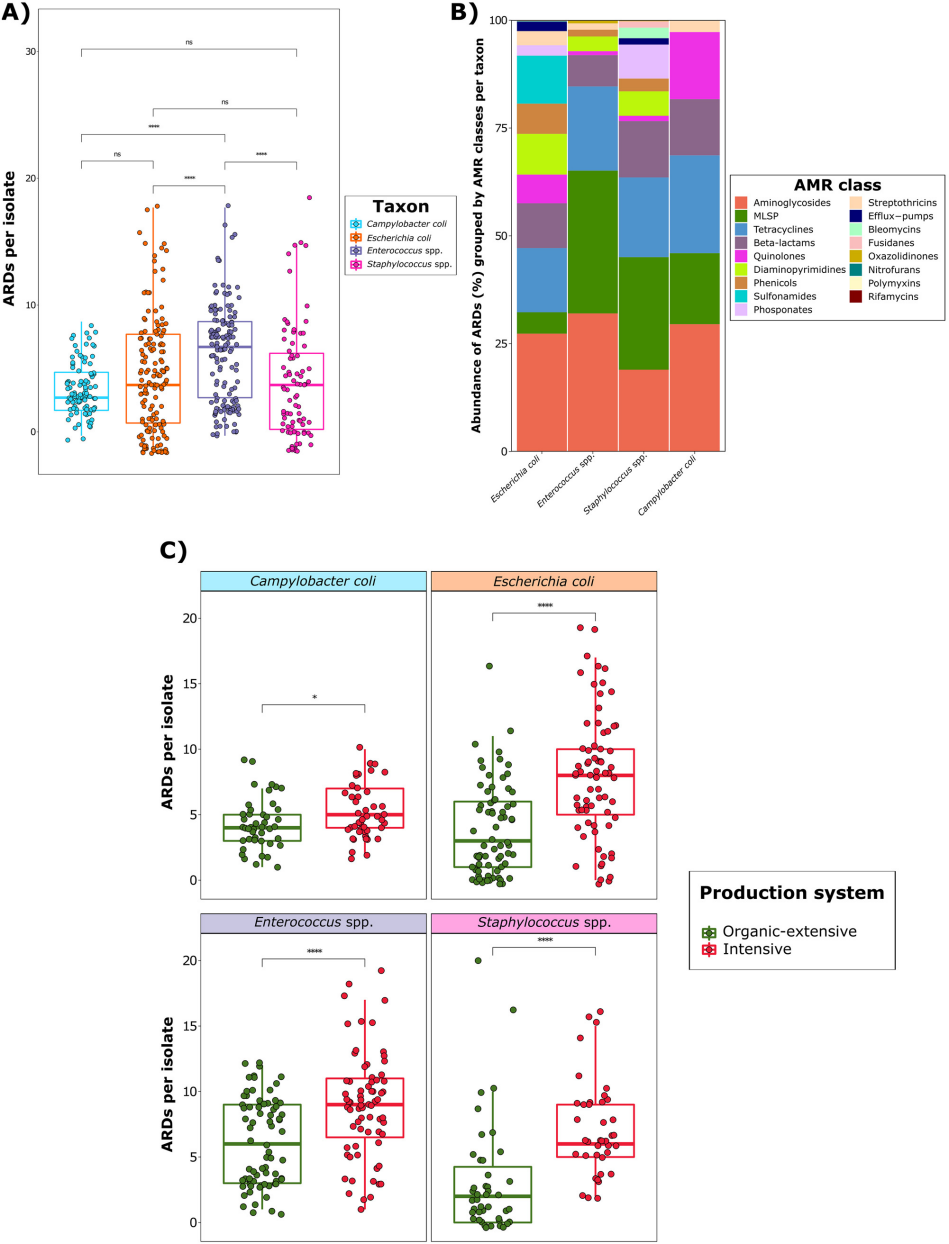
Resistome structure of a collection of isolates from four different bacterial taxa recovered from intensive and organic-extensive farms. (A) Boxplots of total antimicrobial resistance determinants (ARDs) counts per isolate, stratified by taxon. (B) Stacked bar plot of total ARD abundance (expressed as percentages) per antimicrobial resistance (AMR) class (colors) and taxon (*x* axis). (C) Boxplots of total ARD counts per isolate, stratified by production system, within each taxon. Each sample is represented by a dot with horizontal jitter for visibility. Differences per taxon and per production system were evaluated with the Wilcoxon signed-rank test. *n *=* *466 genomes from 37 independent farms belonging to the taxa Campylobacter coli (*n *=* *92), Escherichia coli (*n *=* *145), *Enterococcus* spp. (*n *=* *146), and Staphylococcus spp. (*n *=* *83). MLSP refers to the macrolides-lincosamides-streptogramins-pleuromutilins AMR class. Levels of statistical significance are represented with asterisks: ****, *P* < 0.0001; ***, *P* = 0.0001 to 0.001; **, *P* = 0.001 to 0.01; *, *P* = 0.01 to 0.05; not significant (ns), *P* > 0.05.

A total of 129 unique ARDs, including 96 ARGs and 33 AMR point mutations, from 18 different AMR classes were detected in the isolate collection (Table S1). We observed that the number of ARDs per isolate was significantly higher for *Enterococcus* spp. (*P* < 0.001), with a median of eight (range: 1 to 17), than for any of the other taxa (Fig. 1A). These determinants were predominantly genus-dependent, with only *erm(B)* detected in all taxa (Tables S1 and S2). Nevertheless, Staphylococcus and *Enterococcus* genera, the taxa most closely related phylogenetically in this study, shared 15.6% of their ARGs.

Evaluation of the abundance of ARDs per AMR class within each taxon indicated that the most prevalent AMR classes were aminoglycosides, macrolides-lincosamides-streptogramins-pleuromutilins (MLSP), tetracyclines, and beta-lactams (Fig. 1B). These four classes accounted for most of the AMR diversity, ranging from 57.6% to 91.9% of the total ARDs detected for E. coli and *Enterococcus* spp., respectively. Interestingly, the MLSP AMR class was highly prevalent in all taxa except for E. coli, in which it accounted for only a 5.0% of its resistome. E. coli exhibited the largest AMR diversity, with 60 unique ARDs from 15 different AMR classes; this contrasts with C. coli, which barely harbored 18 unique ARDs belonging to 6 AMR classes. A complete and detailed distribution of ARDs by taxon and isolate is available in the supplemental material files (Fig. S1 to S4 and Tables S1 and S2).

The resistome was clearly influenced by the production system, regardless of genus, with a significantly higher number of ARDs in isolates recovered from intensive herds than in those from organic-extensive herds (Fig. 1C). Analysis of differences at the ARD level revealed that the most frequent ARDs within each genus were significantly more prevalent on the intensive farms than on the organic-extensive ones. This was particularly the case for ARDs which conferred resistance to the most commonly used antimicrobials on swine farms, such as tetracyclines or beta-lactams. All significant differences (*P* < 0.05) across production systems per taxon are shown at the ARD level in the supplemental files (Tables S3 to S6). No substantial differences in ARD distribution were observed in isolates among different sample types (i.e., feces, slurry, farm environment, and oral fluids).

### The ability to predict antimicrobial resistance phenotypes from whole-genome sequencing data varied depending on the taxon.

The AMR genotypes derived from short-read sequencing analyses were compared with the AMR phenotypes of the tested isolates, which had been characterized in previous studies (10, 14). There was an overall good to very good concordance between predicted (WGS-based) and observed (AST-based) susceptibility phenotypes, which was particularly high for E. coli and C. coli, as shown in the supplemental material (Fig. S1 to S4 and Tables S7 to S10). Interestingly, certain ARDs were consistently found in phenotypically susceptible isolates, such as *tet(38)* in tetracycline-susceptible S. aureus isolates and *tet(C)* and *mef(B)* in tetracycline- and azithromycin-susceptible E. coli isolates, respectively.

In Staphylococcus spp., the agreement between predicted and observed phenotypes was less consistent for certain antimicrobials, such as ciprofloxacin or trimethoprim (Table S10A), except for S. aureus, which showed high concordance for most antimicrobials (Table S10B). This result may be attributed to unknown ARDs responsible for the resistant phenotype in poorly characterized Staphylococcus species. For example, in non-*aureus* staphylococci, we detected 14 low-to-medium ciprofloxacin-resistant (MIC: 2 to 4 mg/L) and 17 trimethoprim-resistant (MIC: = 4 to 16 mg/L) isolates with no associated ARD.

### The mobilome is notably diverse, especially in isolates recovered from intensive farms.

The mobilome of the four different taxa consisted of a total of 470 unique MGEs, which included transposable elements (TEs) (71.1%), plasmid incompatibility groups (27.9%), integrons, and prophages (Table S11). TEs (*n *=* *334) were further categorized into composite transposons (CTs) (49.7%), insertion sequences (ISs) (44.3%), and, to a lesser extent, unit transposons (UTs) (0.05%), miniature inverted-repeat transposable elements (MITEs) (0.01%), integrative and conjugative elements (ICEs) (0.005%) and integrative and mobilizable elements (IMEs) (0.003%). These MGEs were taxon-dependent and predominantly found in E. coli, in which we detected 53.4% of all MGE diversity. In contrast, C. coli isolates hardly contributed to the mobilome, with only 5 different MGEs. In fact, we did not detect any MGE in 59.8% of the C. coli isolates, while in Staphylococcus spp. (8.3%), *Enterococcus* spp. (2.1%), and E. coli (0%), most or all isolates harbored MGEs in their genome. A complete and detailed distribution of MGEs by taxon and isolate is available in the supplemental material (Fig. S5 to S8 and Tables S11 and S12).

**FIG 2:**
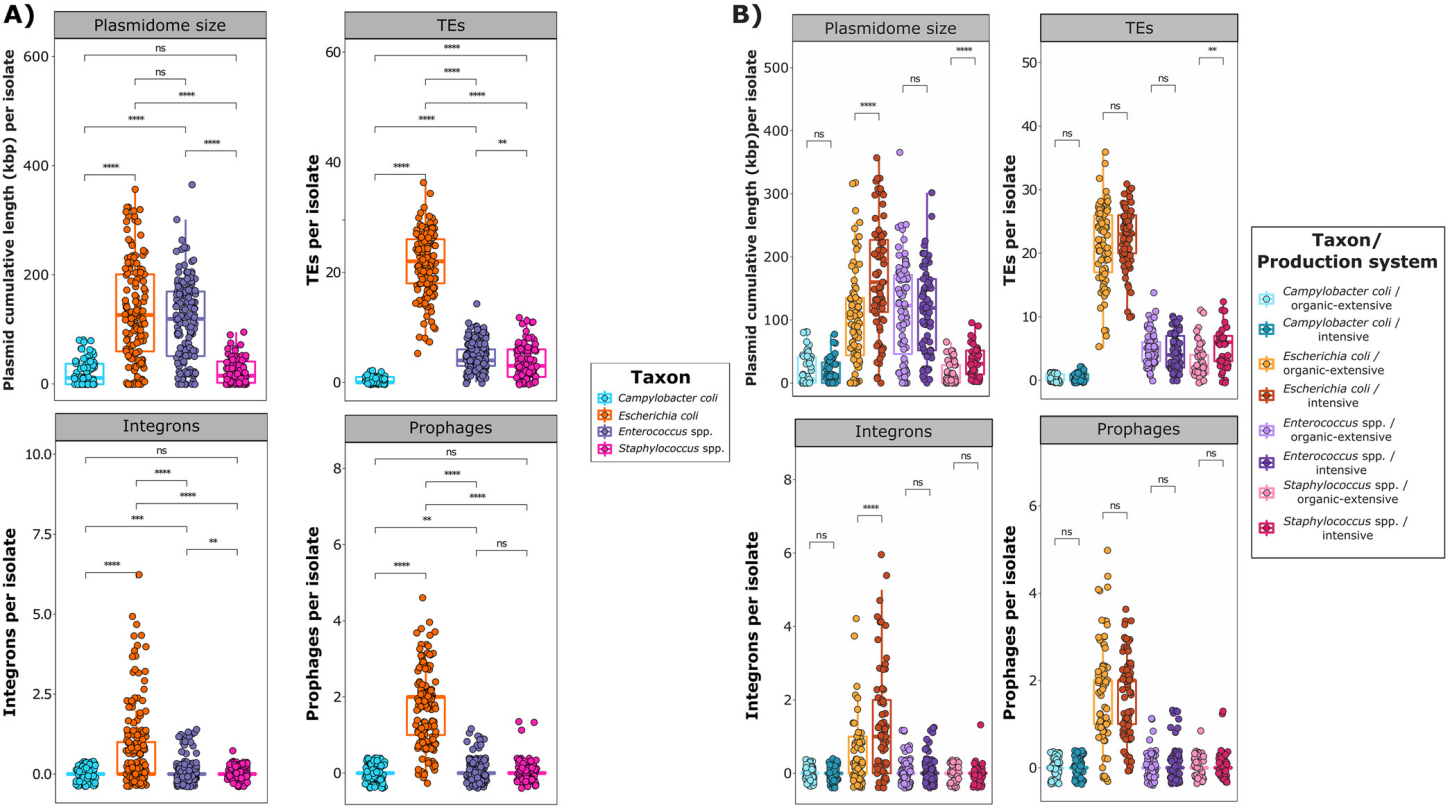
Mobilome composition of a collection of isolates from four different bacterial taxa recovered from intensive and organic-extensive farms. Boxplots of total plasmid content per isolate (kbp), based on the cumulative length of all plasmid-associated contigs, and counts of total transposable elements (TEs), integrons and prophages, stratified by A) taxon, and B) within taxon, by production system. Each sample is represented by a dot with horizontal jitter for visibility. The differences per taxon and per production system were evaluated with the Wilcoxon signed-rank test. *n *=* *466 genomes from 37 independent farms belonging to the taxa Campylobacter coli (*n *=* *92), Escherichia coli (*n *=* *145), *Enterococcus* spp. (*n *=* *146) and Staphylococcus spp. (*n *=* *83). ****, *P* < 0.0001; ***, *P* = 0.0001 to 0.001; **, *P* = 0.001 to 0.01; *, *P* = 0.01 to 0.05; not significant (ns), *P* > 0.05.

TEs were the predominant constituents of the mobilome in E. coli (77.7% of all MGEs), *Enterococcus* spp. (64.3%), and Staphylococcus spp. (58.2%). The number of TEs per isolate was significantly higher in E. coli than in the other taxa (*P* < 0.001), with a median of 22 (range: 5 to 36) (Fig. 2A). Within TEs, this difference was consistently found for ISs, CTs, UTs, and MITEs (Fig. S9). In fact, MITEs were exclusively associated with E. coli and accounted for 27.2% of its mobilome. We observed a significantly lower number of TEs in Staphylococcus spp. isolates (median = 3; range: 0 to 12) than in *Enterococcus* spp. Isolates (median = 4; range: 0 to 14; *P* < 0.01). However, the numbers of CTs and UTs were significantly higher in the former (*P* < 0.01). ICEs and IMEs were the least frequent MGEs but, interestingly, IMEs were significantly more frequent in C. coli (*P* < 0.001) than in any other taxa (Fig. S9).

Plasmidome analyses revealed that total plasmid content, estimated through the cumulative length of all plasmid-associated contigs, was significantly higher in E. coli and *Enterococcus* spp. than in Staphylococcus spp. and C. coli (*P* < 0.001) (Fig. 2A). Remarkably, within *Enterococcus* spp. and Staphylococcus spp., intraspecies differences were also observed. For example, E. faecium had higher plasmid content (*P* < 0.01) than E. faecalis. Staphylococcus spp. isolates showed a greater diversity of plasmid incompatibility groups, despite their reduced plasmid content, compared to E. coli and *Enterococcus* spp. Indeed, in E. coli, we detected 41 unique incompatibility groups, with 2 of them (*ColRNAI_1* and *IncFIB*) accounting for 32.2% of all diversity. In contrast, in Staphylococcus spp., none of the 57 unique incompatibility groups was clearly predominant, highlighting the diversity of the plasmidome within this genus (Fig. S8 and Table S11).

Prophages and integrons were significantly associated with E. coli (*P* < 0.001) (Fig. 2A). Indeed, a total of 258 prophages and 150 integrons were detected, of which 94.2% and 88.0%, respectively, were located in E. coli genomes. Class 1 integrons were predominant in E. coli (39.3% of integrons), followed by class 2 (17.4%) and, to a lesser extent, class 4 (6.8%). *Enterococcus* spp. and Staphylococcus spp. integrons, together with 36.4% of E. coli integrons, could not be characterized at the class level. A detailed description of integrons is available in the supplemental material (Table S13).

The mobilome was influenced by the production system in E. coli and Staphylococcus spp., but not in *Enterococcus* spp. and C. coli (Fig. 2B). Thus, the plasmid cumulative length in isolates recovered from intensive farms was significantly larger for E. coli and Staphylococcus spp. compared to that of isolates from organic-extensive herds (*P* < 0.001). TEs, particularly UTs, were also more frequently associated with Staphylococcus spp. isolates from intensive farms (*P* < 0.05) (Fig. 2B and Fig. S9B). Finally, integrons in E. coli were clearly more abundant among isolates recovered from intensive farms than in those from organic-extensive herds (*P* < 0.001).

### The mobilome plays a differential role in the dissemination of antimicrobial resistance on intensive swine farms.

**FIG 3:**
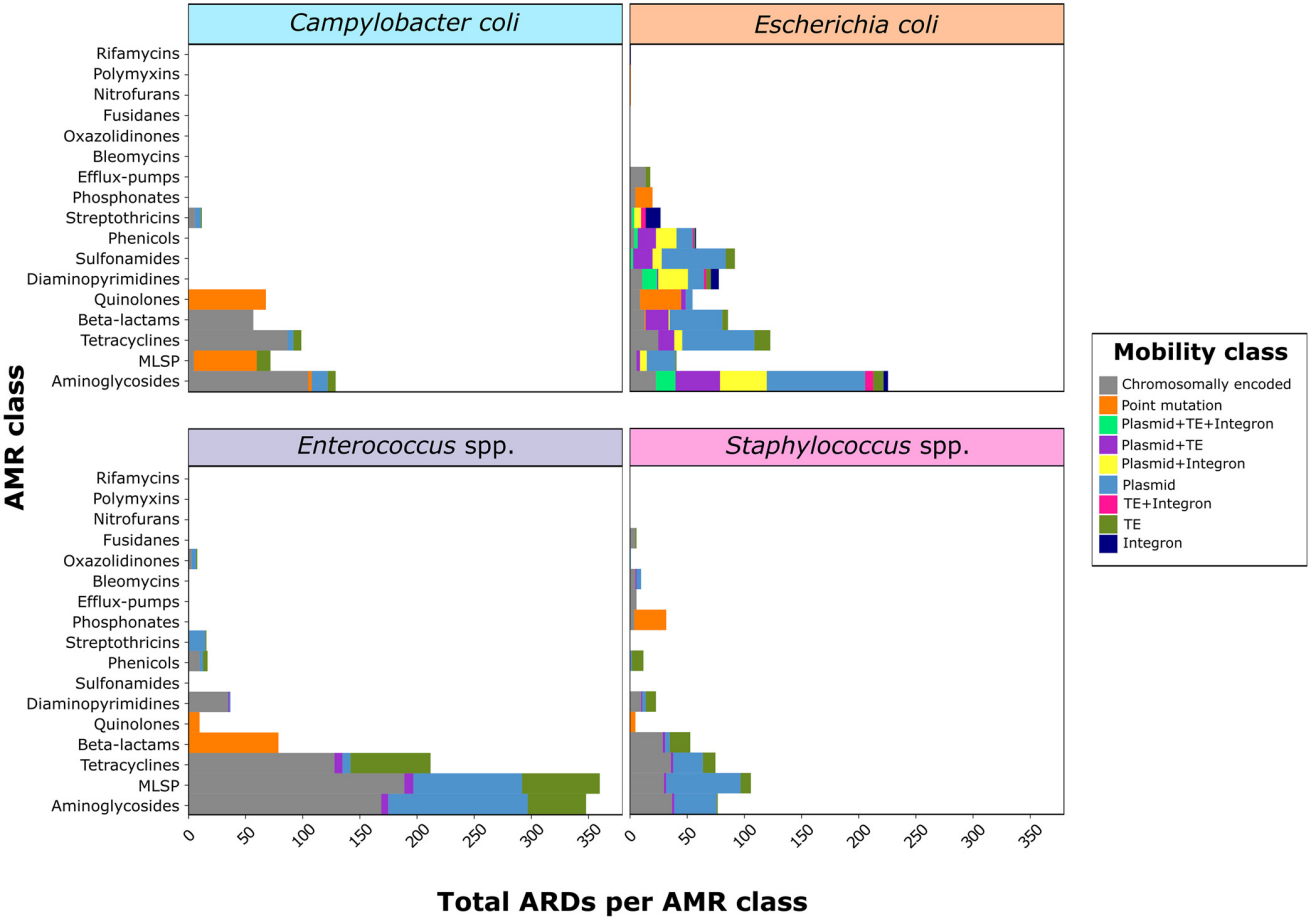
Overview of the association between the resistome and the mobilome in a collection of isolates from four different bacterial taxa. Stacked barplots of antimicrobial resistance determinants (ARDs) counts per mobility class (colors) and per antimicrobial resistance (AMR) class (*y* axis), stratified by taxon. Mobility classes refer to whether the ARD is determined by a point mutation, whether it is chromosomally located or mobilizable and associated with an integron, transposable element (TE), TE + integron, plasmid, plasmid + integron, plasmid + TE or plasmid + TE + integron. *n *=* *466 genomes from 37 independent farms belonging to the taxa Campylobacter coli (*n *=* *92), Escherichia coli (*n *=* *145), *Enterococcus* spp. (*n *=* *146) and Staphylococcus spp. (*n *=* *83). MLSP refers to the macrolides-lincosamides-streptogramins-pleuromutilins AMR class.

The association between resistome and mobilome varied enormously depending on the taxon (Fig. 3). Thus, while mobilizable ARDs, including ARGs located in plasmids and/or those associated with TEs and/or integrons, accounted for 85.4% of all ARDs in E. coli, this association decreased considerably in Staphylococcus spp. (52.9%), *Enterococcus* spp. (43.7%), and C. coli (11.7%). This finding was especially remarkable in *Enterococcus*, the genus which carried the highest number of ARDs, but which were predominantly chromosomally expressed.

In E. coli, the plasmidome was the main reservoir of AMR (harboring 50.3% of all ARDs). ARGs belonging to the most predominant AMR classes, such as aminoglycosides, tetracyclines, or beta-lactams, were significantly more plasmid-associated than chromosome-associated (*P* < 0.001). Interestingly, some of the ARGs detected in E. coli were harbored in TEs and/or integrons located in plasmids. Indeed, TE-associated ARGs were more frequently located in plasmids (18.3%) than in the chromosome (7.3%) in E. coli, inversely to what we found in *Enterococcus* spp. (2.0% versus 17.9%) and Staphylococcus spp. (2.4% versus 14.5%). Although we detected integrons in *Enterococcus* spp. and, to a lesser extent, in Staphylococcus spp., these were not associated with ARG-carrying gene cassettes. In contrast, 58.3% of the integrons in E. coli carried at least one ARG, with a notable role in the dissemination of resistance to aminoglycosides, diaminopyrimidines, and phenicols (Table S13). Interestingly, no prophage was associated with ARDs in any of the taxa. A detailed distribution of AMR classes per taxon and mobility class, together with all significant differences (*P* < 0.05), is shown in the supplemental files (Tables S14 to S21).

**FIG 4:**
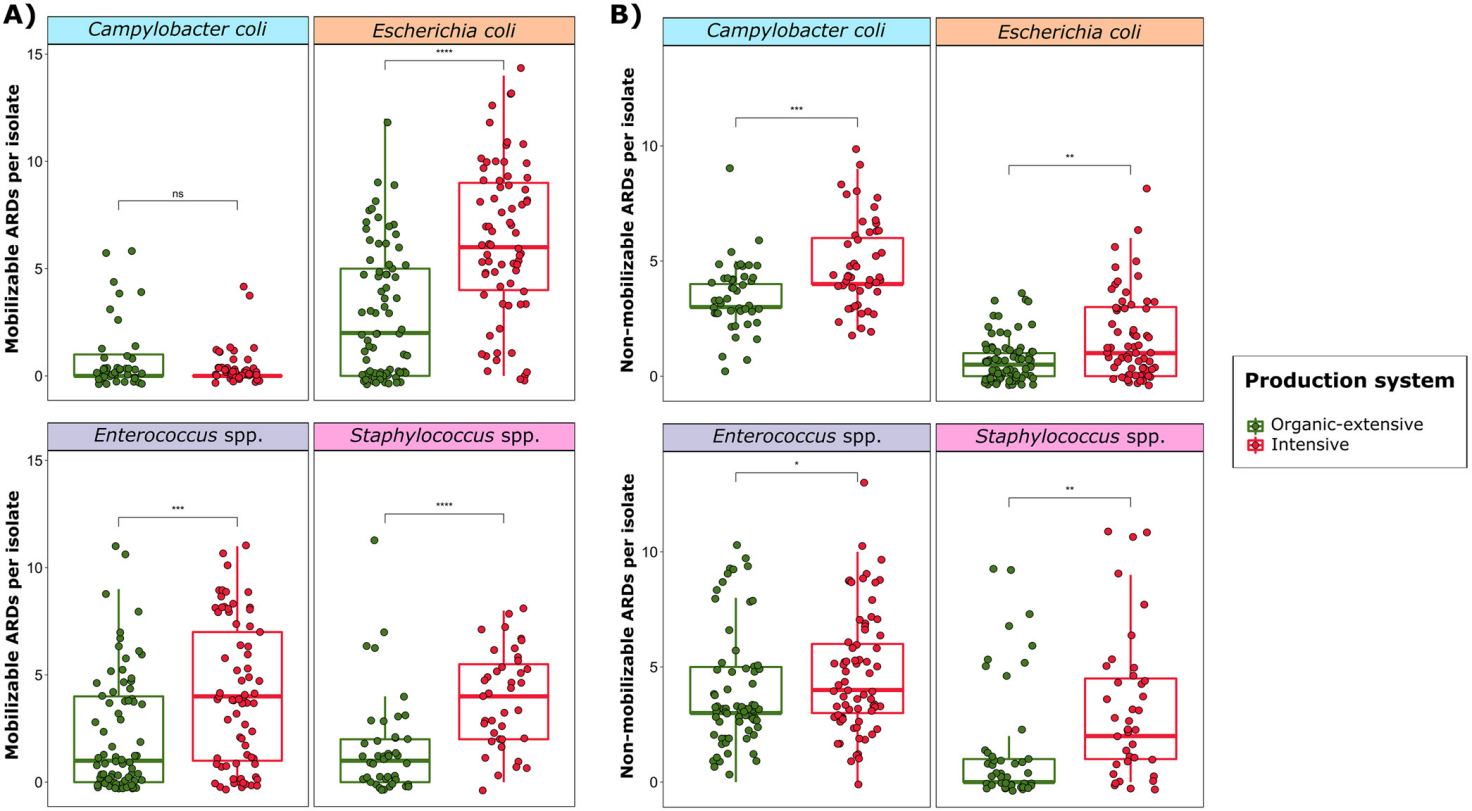
Association between the resistome and the mobilome by production system. Boxplots of total antimicrobial resistance determinants (ARDs) counts per isolate, characterized as A) mobilizable and B) nonmobilizable, stratified by production system within each taxon. Mobilizable ARDs include those antimicrobial resistance genes (ARGs) located in plasmids and/or those associated with transposable elements (TEs) and/or integrons. Nonmobilizable ARDs are referred to point mutations and chromosomal ARGs not associated with TEs and/or integrons. Each sample is represented by a dot with horizontal jitter for visibility. The differences per taxon and per production system were evaluated with the Wilcoxon signed-rank test. *n* = 466 genomes from 37 independent farms belonging to the taxa Campylobacter coli (*n* = 92), Escherichia coli (*n* = 145), *Enterococcus* spp. (*n* = 146) and Staphylococcus spp. (*n* = 83). ****, *P* < 0.0001; ***, *P* = 0.0001 to 0.001; **, *P* = 0.001 to 0.01; *, *P* = 0.01 to 0.05; not significant (ns), *P* > 0.05.

In studying the association between mobilome and resistome by production system, we observed that the number of mobilizable ARDs per isolate was significantly higher in bacteria recovered from intensive farms than in those from organic-extensive farms (*P* < 0.001) for all taxa except for C. coli (Fig. 4A). The lack of difference in C. coli is not surprising considering the reduced number of mobilizable ARDs found in this species. The number of nonmobilizable ARDs, which include point mutations and chromosomally encoded ARGs not associated with TEs and/or integrons, was also significantly higher in bacteria from intensive herds, regardless of taxon (Fig. 4B). However, the associations found were weaker for nonmobilizable than for mobilizable ARDs in *Enterococcus* spp. (*P* < 0.05), E. coli (*P* < 0.01), and Staphylococcus spp. (*P* < 0.01), suggesting that ARG dissemination through MGE mobilization contributes the most to the alarming resistome footprint posed by intensive swine farms.

## DISCUSSION

This study provides a comprehensive insight into the dynamics of the resistome and mobilome of a selection of indicator and zoonotic bacteria recovered from pigs and the environment in a range of swine farms, defining in detail the characteristics of each taxon and potential differences associated with AMU. These results present evidence of the usefulness of characterizing sentinel bacteria, with E. coli and, to a lesser extent, Staphylococcus spp. as the most relevant microorganisms. WGS-based AMR surveillance activities have proven useful in assessing the impact of AMU and AMU-reducing strategies on AMR burden, as revealed by the highly significant differences in the resistome and mobilome, which were associated with sustained differences in AMU across the two swine production systems evaluated.

Following a previously described sampling approach (10, 14), a collection of phylogenetically diverse isolates was obtained, which is consistent with data from similar studies (15–17). We observed several *Enterococcus* and Staphylococcus species and a large phylogenetic diversity in E. coli, particularly compared to C. coli isolates. Multiple STs already linked to livestock and to swine in particular were predominant in our study, including ST-10 in E. coli (18–20), the pig-adapted C. coli ST-854 (21, 22), and livestock-associated MRSA ST-398 among S. aureus strains (23). Overall, the wide genomic diversity observed in the collection of isolates included in this study provides an excellent opportunity for thorough characterization of the resistome and mobilome of these four taxa.

The production system vastly impacted the abundance of ARDs and the composition of the resistome and mobilome for all sentinel bacteria assessed here. Thus, a significantly higher number of ARDs per isolate was observed in bacteria recovered from intensive farms than in those from organic-extensive pig farms, regardless of taxon. Consistently high AMU in intensive herds exerts selective pressure, leading to the perpetuation of AMR bacteria and the consolidation of those ARDs which confer resistance to the antimicrobials most frequently used in these settings (24). When evaluating differences among taxa, *Enterococcus* spp. carried significantly more ARDs per isolate than E. coli, some of which have been described to be intrinsically located in certain *Enterococcus* species (25), such as *aac(6′)-Ia* and *msr(C)* in E. faecium or *dfrE* and *lsa(A)* in E. faecalis; this increases the size of the resistome but poses a limited risk of horizontal AMR dissemination. In contrast, E. coli and, to a lesser extent, Staphylococcus spp. isolates harbored a broader ARD repertoire, mostly consisting of potentially transferable ARGs, which constitute a pressing threat to public health due to to their potential dissemination along the food chain (11). Interestingly, the high prevalence of phenotypic resistance observed in C. coli isolates against the most commonly used antimicrobials for the treatment of human campylobacteriosis was determined by a narrow spectrum of ARDs, with point mutations in chromosomal genes as the main AMR drivers, in agreement with previous studies (17, 26). Thus, both indicator and zoonotic bacteria were shown to be important AMR reservoirs, with remarkable qualitative and quantitative differences in the ARDs they harbored.

HGT events are key elements for the mobilization of ARDs from the bacterial chromosome to transferable MGEs, such as plasmids or TEs (27). The complex networks formed by these MGEs have the potential to recruit and disseminate genes throughout bacterial communities, driving the spread of AMR (2). In this study, the structure of the mobilome in the evaluated bacteria was predominantly conditioned by taxonomic affiliation, evidencing its phylogenetic adaptation (4, 28, 29). Moreover, the differential AMU observed between both production systems clearly influenced the mobilome and, in particular, the abundance and diversity of mobilizable ARDs, except for in C. coli isolates, a result which confirms the findings of a previous metagenomics study carried out on these farms (8).

The mobilome was particularly complex in E. coli, with a great diversity of TEs identified, suggesting that MGE transposition is relatively common in this species (30). The plasmidome, considered to be a determinant for AMR dissemination (31), was also especially relevant in terms of ARD carriage in E. coli genomes, evidencing its role in the AMR mobilization (15, 32). Indeed, most of the constituents of the E. coli resistome were predicted to be associated with MGEs, and about half of them, with plasmids, either exclusively or coupled with TEs and/or integrons. These findings demonstrate that, among the assessed sentinel microorganisms, E. coli represented the largest reservoir of mobilizable ARGs, and suggest that consistently high AMU promotes and enhances AMR dissemination in this species. In contrast, even though C. coli is considered to be the Campylobacter species which harbors the highest number of plasmid-associated ARGs (17), the relevance of the plasmidome in AMR dissemination for this species was residual in this study. Nevertheless, it is important to bear in mind that HGT in C. coli is not exclusively restricted to conjugation because transduction and, in particular, natural transformation are essential in the transference of chromosomal ARDs, including point mutations against critically important antimicrobials, such as macrolides or quinolones (33).

It has been described that *Enterococcus* spp. can act as a hub for MGEs, disseminating ARDs among both Gram-positive and Gram-negative bacteria, most notably to Staphylococcus spp. and Streptococcus spp. (34). Although in this study the *Enterococcus* spp. plasmidome was similar in size to that of E. coli, the complexity of the interactions between mobilome and resistome was much lower for *Enterococcus* spp. The reduced number of plasmid-associated ARGs observed in *Enterococcus* spp. isolates suggests a limited AMR dissemination from swine farm isolates to their nosocomial counterparts, particularly in E. faecium, the most predominant species in this study, in which the plasmidome has been revealed as the main genomic component driving niche specificity (16, 35). In contrast, the generalist nature of E. faecalis enables the survival and spread of AMR strains across multiple niches, such as hospital environments and the human gut (34, 36), resulting in a greater threat to human health. In *Enterococcus* spp. isolates, most ARGs were chromosomally located, with no presumptive association with TEs. Since most of these nonmobilizable ARGs are not part of the *Enterococcus* core genome, it is suggested that these must have been transferred at some earlier point to the bacterial chromosome and, therefore, most of them should be associated with putative TEs. In addition, the inference approach used in this study to detect large TEs has limited accuracy when these cannot be fully assembled in context using short-read sequencing, resulting in an underrepresentation of MGEs (2, 30). Indeed, a recent study revealed that rare, and hence uncharacterized, ISs are common in *Enterococcus*, particularly, in E. faecium (30). This finding may also be extended to Staphylococcus spp. isolates, since most of them belonged to so far poorly characterized species. The large number of Staphylococcus species characterized in this study might further explain the huge plasmidome diversity, which was remarkably higher than that found for the other sentinel microorganisms. In conclusion, although the mobilome of isolates from these two genera, and particularly of certain species, such as E. faecium, was not strongly associated with the resistome on swine farms, further studies should be conducted to characterize currently unknown associations between putative TEs and chromosomal ARGs.

In the present study, we observed a good concordance between observed (AST-based) and predicted (WGS-based) AMR phenotypes, which was particularly high in E. coli and C. coli, in agreement with previous studies (19, 26); but not in *Enterococcus* spp. and Staphylococcus spp., especially in those species with less epidemiological and pathogenic relevance. The accuracy of databases, the presence of unknown AMR mechanisms, and mismatches associated with gene expression, which can result in overexpression or repression of the related resistance phenotypes, are factors which may influence the level of concordance reached (37). In addition, with short-read genomic assembly, there can be duplicated ARDs in the same strain which could be detected only once, which may explain disagreements with the observed AMR phenotypes. Overall, although AST is the gold standard method in AMR surveillance, WGS offers a good prediction of AMR phenotypes while also providing detailed information on their determinants and genomic background. However, further research is needed to overcome potential disagreements, especially in poorly characterized bacteria that are not usually included in AMR surveillance schemes, for which less information on AMR mechanisms is available from AMR databases.

The detailed characterization of sentinel microorganisms using a short-read WGS approach provides several benefits for AMR surveillance, as evidenced in the current study, since it enables a comprehensive analysis of the resistome and its associated mobilome. The monitoring of these microorganisms, particularly E. coli and, to a lesser extent, Staphylococcus spp., could be used as a rapid and efficient technique for the assessment of mid- to long-term on-farm interventions for AMU reduction and the evaluation of the AMR status. However, although the short-read WGS approach used in this study provides an overview of the overall AMR and MGE burden, to overcome the aforementioned limitations of this technique and provide deeper characterization of the resistome-mobilome interaction, the use of a hybrid assembly based on a combination of short- and long-read sequencing would produce complete, high-quality genomes, allowing for accurate structural resolution (38).

To the best of our knowledge, this study provides the first integral genomic characterization of the resistome and mobilome of sentinel microorganisms for AMR surveillance on swine farms. Significant differences were observed among taxa in the ARDs and MGEs they harbored, revealing the distinctive risks they pose in AMR dissemination and spread. Finally, the inclusion of farms with significant differences in AMU allowed us to assess the suitability of WGS of these sentinel taxa for monitoring AMR trends, showing that on farms with lower AMU, the quantitative composition of the resistome was significantly lower in all taxa, as was the complexity of the resistome-mobilome interactions. Overall, this study demonstrates that responsible AMU can contribute to reducing AMR pressure in the food chain.

## MATERIALS AND METHODS

### Sampling and bacterial isolation and phenotypic characterization.

A total of 37 swine farms spread across the Spanish geography were selected according to their production system to provide a representative sample of Spanish intensive (18 herds), extensive (12 herds) and organic (7 herds) production systems. Organic and extensive farms were merged into a single category because organic herds were mainly converted from extensive farms rearing Iberian pigs on a system based on the use of natural resources in farrow-to-finish farms. Thus, farms were grouped into intensive (18 herds) and organic-extensive (19 herds) for further analyses.

Sampling, farm characteristics, bacterial isolation data, and AST data have been detailed by Mencía-Ares et al. (10, 14). Briefly, samplings were conducted in 2017 and 2018 in pigs during the last month of the fattening period, with no antimicrobial treatment in the month prior to sampling. In each fattening unit, feces, environmental swabs, ropes with oral fluids, and slurry, when available, were collected. While Escherichia coli, *Enterococcus* spp. and Campylobacter coli were isolated from feces, slurry and environmental samples, Staphylococcus spp. were isolated from environmental and oral fluid samples. AST was conducted using the procedures outlined by the European Committee on Antimicrobial Susceptibility Testing (EUCAST) (39).

### Genomic DNA extraction and whole-genome sequencing.

E. coli, *Enterococcus* spp., and Staphylococcus spp. were initially cultured on brain heart infusion (BHI) agar (Merck, Darmstadt, Germany) at 37°C for 24 h, while C. coli was initially grown on fastidious anaerobe agar (Neogen, Heywood, United Kingdom) at 41.5°C for 48 h. A single colony from each pure culture was inoculated in BHI broth (Merck) and grown under continuous agitation (180 to 220 rpm) at 37°C for 24 h, except for C. coli, which was incubated at 41.5°C for 48 h. All C. coli incubations were performed under microaerophilic conditions using CampyGen sachets (Oxoid, Basingstoke, United Kingdom).

Genomic DNA was extracted from liquid cultures using the PureLink Genomic DNA minikit (Invitrogen, Carlsbad, CA, USA) following the manufacturer’s protocols for Gram-negative (E. coli and C. coli) and Gram-positive (*Enterococcus* spp. and Staphylococcus spp.) bacteria. Prior to sequencing, a Qubit dsDNA BR Assay (Invitrogen, Eugene, OR, USA) was used to determine the DNA concentration. Sequencing libraries were prepared with the Nextera DNA Flex Library Preparation kit (Illumina, San Diego, CA, USA) according to the manufacturer’s guidelines. Paired-end sequencing was performed on the Illumina NextSeq 500 benchtop sequencer using the NextSeq 500/550 Mid Output kit v2.5 (300-cycle). DNA sequences from the 466 isolates are publicly available at the Sequence Read Archive database under NCBI ID no. PRJNA776103.

### Genomic assembly and bacterial characterization.

Raw reads were trimmed and *de novo* assembled with the AQUAMIS pipeline v1.2.0 (40), which implements fastp v0.19.5 for trimming (41), shovill v1.1.0 (https://github.com/tseemann/shovill) for assembly, automated reference search of complete NCBI RefSeq genomes using Mash v2.2.2, (42) and assembly quality analysis using Quast v5.0.2 (https://github.com/ablab/quast). Based on the draft assemblies produced by AQUAMIS, bacterial genomes were characterized using the BakCharak pipeline v1.0.0 (https://gitlab.com/bfr_bioinformatics/bakcharak). This pipeline runs NCBI AMRFinder v3.8.4 (database v2021-03-01.1) (43) to detect ARDs, ABRicate v1.0.1 (https://github.com/tseemann/abricate) to detect plasmid incompatibility groups from the PlasmidFinder database (44), Platon v1.4.0 (45) to identify and characterize plasmid contigs and Prokka v1.14.6 (46) for genome annotation. Moreover, genomic phylogenetic characterization was performed by computing ANI between all samples of a taxon using FastANI v1.31 (47) and a seven-gene MLST conducted by mlst v2.19.0 (https://github.com/tseemann/mlst) and the PubMLST schemes (48) for species with an MLST scheme available.

The tool MobileElementFinder v1.0.3 (2)—using default parameters—was used to predict TEs, which were further categorized into ISs, CTs, UTs, MITEs, IMEs and ICEs. Based on the genomes’ Prokka annotation, putative prophages were classified using PhiSpy v4.1.6 (49), in conjunction with the pVOG database (50). IntegronFinder v2-2021-09-22 (51) was used to detect and characterize integrons in the assembled genomes. Integron characterization was subsequently performed by a BLASTx (52) of contigs harboring integrons versus a database containing aminoacidic sequences of integrases from type 1 (WP_000845048.1), type 2 (WP_063962748.1), type 3 (WP_013250880.1) and type 4 (QID23267.1). Only those hits with a percentage of identity higher than 70% were kept for further analyses. Integrons were classified according to the best BLAST hit obtained. ARG annotation of the integrons was performed using the hidden Markov model (HMM) from the AMRfinder database v3.8.4.

### Curation of antimicrobial resistance determinants.

The AMRFinder output included both ARGs and point mutations, which were unified and defined as ARDs. Certain ARGs were removed from the study due to a lack of consensus regarding their roles in AMR, including *blaEC*, *acrF*, *mdtM*, and *eat(A)*, along with transcriptional regulators of ARGs, such as *blaI*, *blaR1*, and *mecR1*. In addition, some ARGs were renamed to maintain the same nomenclature and avoid duplications. Thus, we renamed *ant(3″)-Ia* to *aadA1*, *blaPC1* to *blaZ*, *aadE* to *ant(6)-Ia*, and *aad9*, *spw*, and *spd* to *ant(9)-Ia*.

The document “phenotypes.txt” from the ResFinder repository (2021-05-03) (https://bitbucket.org/genomicepidemiology/resfinder_db/src/master/), together with documents from the PointFinder repository (2021-05-03) (https://bitbucket.org/genomicepidemiology/pointfinder_db/src/master/) for E. coli, Campylobacter, E. faecium, E. faecalis, and S. aureus, were downloaded to manually curate the AMRFinder output, and thereby extrapolate the AMR genotype for each isolate at class and antimicrobial level. Thus, we modified the “class” variable, gathering ARDs that confer resistance to macrolides, lincosamides, streptogramins, and pleuromutilins into the MLSP class and those that confer resistance to oxazolidinones into the oxazolidinones class. This last group included *cfr* genes, which confer resistance to phenicols, lincosamides, oxazolidinones, pleuromutilins, and streptogramins A, the *optrA* gene, which confers resistance to phenicols and oxazolidinones, and the *poxtA* gene, which confers reduced susceptibility to phenicols, oxazolidinones, and tetracyclines.

### Characterization of the resistome-mobilome association.

To establish the association between ARDs and MGEsm we parsed and combined the results from different tools. First, when an ARD was located on a contig that had been previously classified as plasmid by Platon, the ARD was considered mobilizable and plasmid-associated. All ARDs which had been annotated within the integrons detected by IntegronFinder were considered mobilizable and integron-associated. Moreover, if a prophage region was predicted by PhiSpy and an ARD was found in the same region, the ARD was also considered mobilizable and prophage-associated. Finally, if an ARD was on the same contig as a TE—based on MobileElementFinder results—the ARD was considered mobilizable and TE-associated if the ARD was located either within the TE (e.g., in the case of a CT) or within a maximum distance of 31,000 bp from a TE (e.g., co-located with an IS). This maximum distance, TE-ARG, was set considering the findings of a previous study (2). Note that ARDs may not be flanked by, for instance, two ISs, as contig breaks are frequent given the presence of repeat-sequences in this region. Overall, ARDs could be classified as mobilizable when they were associated with one or more MGEs. In contrast, nonmobilizable ARDs were referred to point mutations and chromosomal ARGs not associated with TEs and/or integrons.

### Statistical analysis and figures visualization.

Analyses were performed among the four different taxa (E. coli, *Enterococcus* spp., Staphylococcus spp. and C. coli) and, within each taxon, among the two production systems (intensive and organic-extensive) and the four sample types (environment, feces, oral fluids and slurry). Differential analyses were also conducted among the most predominant species in *Enterococcus* spp. (E. faecium and E. faecalis) and Staphylococcus spp. (*S. haemolyticus*, *S. chromogenes*, *S. hyicus* and S. aureus). All analyses were performed using R v4.0.5 (53).

The differential abundance of ARDs and MGEs among the study variables (taxon, production system and sample type) was estimated using the Wilcoxon signed-rank test through the ggpubr package v0.4.0 (54). All *P* values were adjusted by following the Benjamini and Hochberg method (55) and significance was established at *P* < 0.05. In addition, to assess which ARDs and MGEs were shared between taxa and evaluate how these were distributed by production system and sample type within each taxon, we used the “*setdiff*” function in dplyr package v1.0.5 (56). Within-taxon dissimilarities of STs, ARDs, and MGEs at production system and sample type level were analyzed using Fisher’s exact test. This test was also used to evaluate the mobilome-resistome association within each taxon, particularly focusing on the differential distribution among antimicrobial classes.

The association between phenotypic (broth microdilution AST-based) and genotypic (WGS-based) AMR was assessed by evaluating the concordance between the carriage of ARDs with known resistance outcomes and the expression of an antimicrobial resistant phenotype. ARDs associated with an unclear or reduced susceptibility phenotype, including the *poxtA* and *msr(C)* genes and the *50S_L22_A103V*, *pbp5*, *parC* and *parE* point mutations, were excluded from the analysis. Sensitivity and specificity of WGS-based predictions were calculated for each antimicrobial tested using the corresponding phenotypic reference. Inter-rater agreement analyses were performed for each antimicrobial using Cohen’s kappa (κ), including its 95% confidence interval (95% CI). Interpretation of κ values to assess the strength of agreement between techniques was based on the method proposed by Altman (57), which is as follows: κ ≤ 0.20 = poor; 0.21 to 0.40 = fair; 0.41 to 0.60 = moderate; 0.61 to 0.80 = good; and 0.81 to 1.00 = very good.

The resistome and the mobilome per taxon were summarized in heatmaps with dendrograms constructed with the ComplexHeatmap package v2.6.2 (58) in combination with the dendextend package v1.14.0 (59). Dendrograms represented the 100-ANI clustering of isolates belonging to the same taxon. Isolates showing ≤76% ANI among themselves were assigned a value of 75% to enable visualization of the complete dendrogram. Resistome heatmaps were constituted by combining the AMR genotype, represented by the presence-absence matrix of ARDs, and the AMR phenotype, given by a standardized MIC gradient of the antimicrobials tested, all also grouped by antimicrobial class. Mobilome heatmaps depicted the entire set of MGEs detected per isolate and grouped by mobility class. Other plots were produced using the ggplot2 package v3.3.3 (60), and further modified using the software Inkscape v1.1.1 (https://inkscape.org/).
