# Supplementary material for: Genomic Insights into the Mobilome and Resistome of Sentinel Microorganisms Originating from Farms of Two Different Swine Production Systems
Source: Microbiol Spectr. 2022 Nov 15;10(6):e02896-22. doi: 10.1128/spectrum.02896-22 (PMC9769681; doi:10.1128/spectrum.02896-22)
Supplement: Supplemental file 10 — Fig. S1 to S9. Download spectrum.02896-22-s0010.pdf, PDF file, 1.4 MB [file spectrum.02896-22-s0010.pdf]

Supplemental material

Figure S1. Comprehensive summary of the antimicrobial resistance (AMR) phenotype and genotype per isolate in 145 *Escherichia coli* recovered from intensive and organic-extensive pig farms. Dendrograms represented the 100-ANI phylogenetic clustering of the isolates. Sequence type (ST) for each isolate was included when available. AMR genotype was represented by the presence-absence of antimicrobial resistance determinants (ARDs), grouped by AMR class. AMR phenotype was given by a standardized minimum inhibitory concentration (MIC) gradient of the antimicrobials tested, also grouped by AMR class. The production system and the source of the isolate are specified by different colors. MLSP refers to the macrolides-lincosamides-streptogramins-pleuromutilins AMR class.

Figure S2. Comprehensive summary of the antimicrobial resistance (AMR) phenotype and genotype per isolate in 146 *Enterococcus* spp. recovered from intensive and organic-extensive pig farms. Dendrograms represented the 100-ANI phylogenetic clustering of the isolates. Isolates from different species showing $\leq$ 76% ANI among themselves were assigned a value of 75% to enable the visualization of the complete dendrogram. Sequence type (ST) for each isolate was included when available. AMR genotype was represented by the presence-absence of antimicrobial resistance determinants (ARDs), grouped by AMR class. AMR phenotype was given by a standardized minimum inhibitory concentration (MIC) gradient of the antimicrobials tested, all also grouped by AMR class. The production system, the source of the isolate and the *Enterococcus* species are specified by different colors. MLSP refers to the macrolides-lincosamides-streptogramins-pleuromutilins AMR class.

Figure S3. Comprehensive summary of the antimicrobial resistance (AMR) phenotype and genotype per isolate in 92 *Campylobacter coli* recovered from intensive and organic-extensive pig farms. Dendrograms represented the 100-ANI phylogenetic clustering of the isolates. Sequence type (ST) for each isolate was included when available. AMR genotype was represented by the presence-absence of antimicrobial resistance determinants (ARDs), grouped by AMR class. AMR phenotype was given by a standardized minimum inhibitory concentration (MIC) gradient of the antimicrobials tested, also grouped by AMR class. The production system and the source of the isolate are specified by different colors. MLSP refers to the macrolides-lincosamides-streptogramins-pleuromutilins AMR class.

Figure S4. Comprehensive summary of the antimicrobial resistance (AMR) phenotype and genotype per isolate in 83 *Staphylococcus* spp. recovered from intensive and organic-extensive pig farms. Dendrograms represented the 100-ANI phylogenetic clustering of the isolates. Isolates from different species showing ≤ 76% ANI among themselves were assigned a value of 75% to enable the visualization of the complete dendrogram. Sequence type (ST) for each isolate was included when available. AMR genotype was represented by the presence-absence of antimicrobial resistance determinants (ARDs), grouped by AMR class. AMR phenotype was given by a standardized minimum inhibitory concentration (MIC) gradient of the antimicrobials tested, also grouped by AMR class. The production system, the source of the isolate and the *Staphylococcus* species are specified by different colors. MLSP refers to the macrolides-lincosamides-streptogramins-pleuromutilins AMR class.

Figure S5. Comprehensive summary of mobile genetic elements (MGEs) detected per isolate in 145 *Escherichia coli* recovered from intensive and organic-extensive pig farms. Dendrograms represented the 100-ANI phylogenetic clustering of the isolates.

The production system and the source of the isolate are specified by different colors. The mobilome was represented by the number of unique MGEs detected per isolate, which were also grouped by mobility class. These include plasmid incompatibility groups, prophages, integrons and transposable elements (TEs), which were further grouped into composite transposons (CTs), insertion sequences (ISs), miniature inverted-repeat transposable elements (MITEs) and unit transposons (UTs).

Figure S6. Comprehensive summary of mobile genetic elements (MGEs) detected per isolates in 146 *Enterococcus* spp. recovered from intensive and organic-extensive pig farms. Dendrograms represented the 100-ANI phylogenetic clustering of the isolates. Isolates from different species showing ≤ 76% ANI among themselves were assigned a value of 75% to enable the visualization of the complete dendrogram. The production system, the source of the isolate and the *Enterococcus* species are specified by different colors. The mobilome was represented by the number of unique MGEs detected per isolate, which were also grouped by mobility class. These include plasmid incompatibility groups, prophages, integrons and transposable elements (TEs), which were further grouped into composite transposons (CTs), integrative and conjugative elements (ICEs), integrative and mobilizable elements (IMEs), insertion sequences (ISs) and unit transposons (UTs).

Figure S7. Comprehensive summary of mobile genetic elements (MGEs) detected per isolate in 92 *Campylobacter coli* recovered from intensive and organic-extensive pig farms. Dendrograms represented the 100-ANI phylogenetic clustering of the isolates. The production system and the source of the isolate are specified by different colors. The mobilome was represented by the number of unique MGEs detected per isolate, which were also grouped by mobility class. These include plasmid incompatibility groups and

transposable elements (TEs), which were further grouped into integrative and mobilizable elements (IMEs) and insertion sequences (ISs).

Figure S8. Comprehensive summary of mobile genetic elements (MGEs) detected per isolate in 83 *Staphylococcus* spp. recovered from intensive and organic-extensive pig farms. Dendrograms represented the 100-ANI phylogenetic clustering of the isolates. Isolates from different species showing ≤ 76% ANI among themselves were assigned a value of 75% to enable the visualization of the complete dendrogram. The production system, the source of the isolate and the *Staphylococcus* species are specified by different colors. The mobilome was represented by the number of unique MGEs detected per isolate, which were also grouped by mobility class. These include plasmid incompatibility groups, prophages, integrons and transposable elements (TEs), which were further grouped into composite transposons (CTs), integrative and conjugative elements (ICEs), insertion sequences (ISs) and unit transposons (UTs).

Figure S9. Itemization of transposable elements (TEs) composition in bacteria from four different taxa recovered from intensive and organic-extensive farms. Boxplots of composite transposons (CTs), insertion sequences (ISs), integrative and conjugative elements (ICEs), integrative and mobilizable elements (IMEs), miniature inverted-repeat transposable elements (MITEs) and unit transposons (UTs) counts per isolate, stratified by A) taxon and, within taxon, by B) production system. Each sample is represented by a dot with horizontal jitter for visibility. The differences per taxon and per production system were evaluated with the Wilcoxon signed-rank test. n = 466 genomes from 37 independent farms belonging to the taxa *Campylobacter coli* (n = 92), *Escherichia coli* (n = 145), *Enterococcus* spp. (n = 146) and *Staphylococcus* spp. (n = 83).

Table S1. Curated summary of ARDs detected on the collection of isolates sequenced, including their distribution per taxon. MLSP refers to the macrolides-lincosamides-streptogramins-pleuromutilins AMR class.

Table S2. Distribution of ARDs per taxon.

Table S3. Differential distribution of ARDs in *Escherichia coli* between production systems ($p < 0.05$) by Fisher's exact test, including ARD counts for each group.

Table S4. Differential distribution of ARDs in *Enterococcus* spp. between production systems ($p < 0.05$) by Fisher's exact test, including ARD counts for each group.

Table S5. Differential distribution of ARDs in *Campylobacter coli* between production systems ($p < 0.05$) by Fisher's exact test, including ARD counts for each group.

Table S6. Differential distribution of ARDs in *Staphylococcus* spp. between production systems ($p < 0.05$) by Fisher's exact test, including ARD counts for each group.

Table S7. Concordance tests between phenotypic AST and WGS-based AMR predictions in *Escherichia coli*.

Table S8. Concordance tests between phenotypic AST and WGS-based AMR predictions in A) *Enterococcus* spp., B) *E. faecium* and C) *E. faecalis*.

Table S9. Concordance tests between phenotypic AST and WGS-based AMR predictions in *Campylobacter coli*.

Table S10. Concordance tests between phenotypic AST and WGS-based AMR predictions in A) *Staphylococcus* spp., B) *S. aureus*, C) *S. haemolyticus*, D) *S. chromogenes* (D) and E) *S. hyicus*.

Table S11. Curated summary of MGEs detected on the collection of isolates sequenced, including their distribution per taxon.

Table S12. Distribution of MGEs per taxon.

Table S13. Detailed summary of 151 integrons detected on the collection of isolates sequenced.

Table S14. Detailed distribution by A) mobility class and by B) grouped mobility class of total ARD counts and ARD counts split by AMR class in *Escherichia coli*, expressed as a percentage.

Table S15. Detailed distribution by A) mobility class and by B) grouped mobility class of total ARD counts and ARD counts split by AMR class in *Enterococcus* spp., expressed as a percentage.

Table S16. Detailed distribution by mobility class of total ARD counts and ARD counts split by AMR class in *Campylobacter coli*, expressed as a percentage.

Table S17. Detailed distribution by A) mobility class and by B) grouped mobility class of total ARD counts and ARD counts split by AMR class in *Staphylococcus* spp., expressed as a percentage.

Table S18. Differential distribution of ARDs split by AMR class in *Escherichia coli* among A) mobility classes and B) grouped mobility classes ($p < 0.05$) by Fisher's exact test, including ARD counts for each group.

Table S19. Differential distribution of ARDs split by AMR class in *Enterococcus* spp. among A) mobility classes and B) grouped mobility classes ($p < 0.05$) by Fisher's exact test, including ARD counts for each group.

Table S20. Differential distribution of ARDs split by AMR class in *Campylobacter coli* among mobility classes ($p < 0.05$) by Fisher's exact test, including ARD counts for each group

Table S21. Differential distribution of ARDs split by AMR class in *Staphylococcus* spp. among A) mobility classes and B) grouped mobility classes ($p < 0.05$) by Fisher's exact test, including ARD counts for each group.

Figure S1

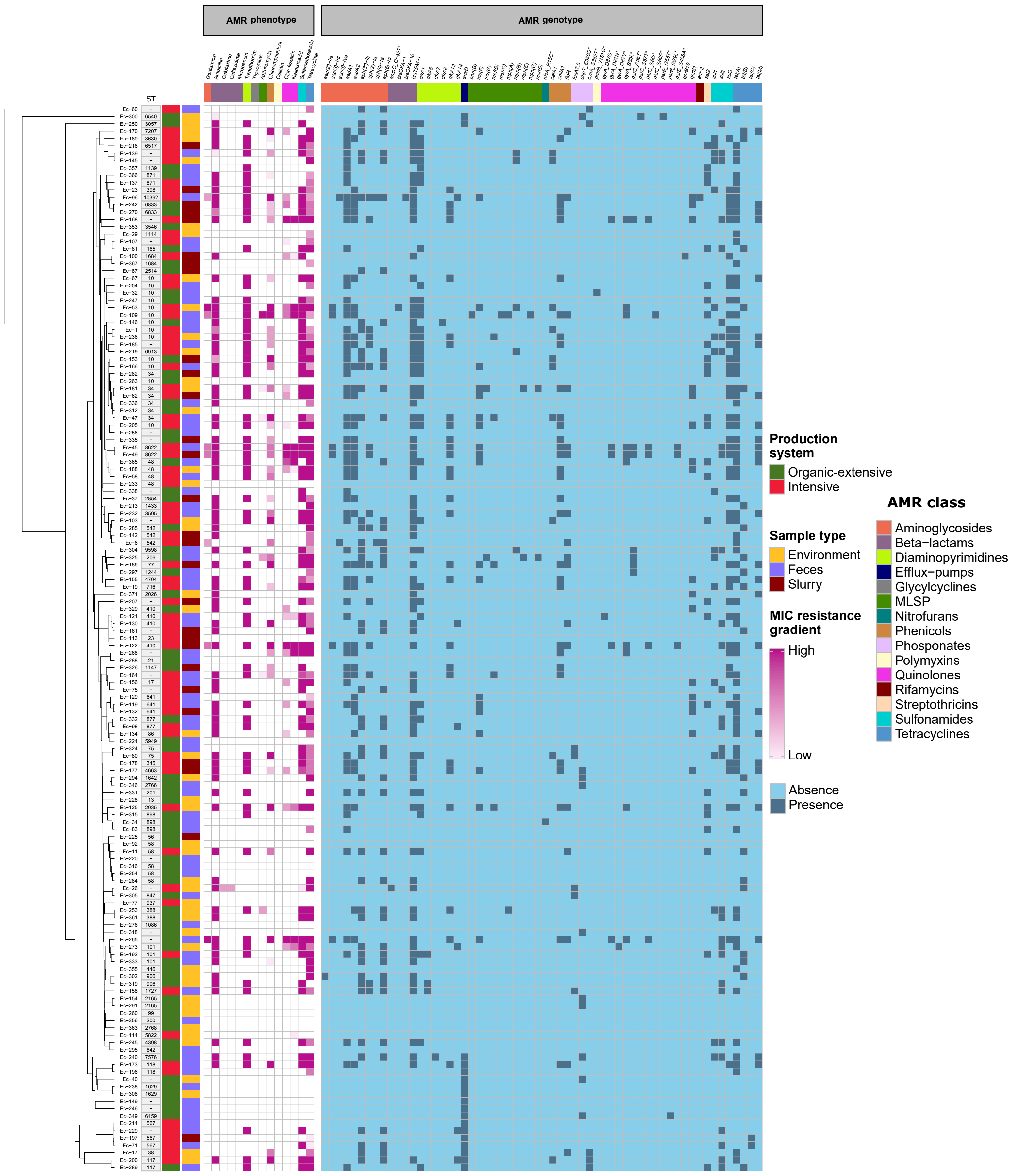
AMR phenotype
AMR genotype
ST
Production system
Organic-extensive
Intensive
Sample type
Environment
Feces
Slurry
MIC resistance gradient
High
Low
Absence
Presence
AMR class
Aminoglycosides
Beta−lactams
Diaminopyrimidines
Efflux−pumps
Glycylcyclines
MLSP
Nitrofurans
Phenicols
Phosponates
Polymyxins
Quinolones
Rifamycins
Streptothricins
Sulfonamides
Tetracyclines

Figure S2

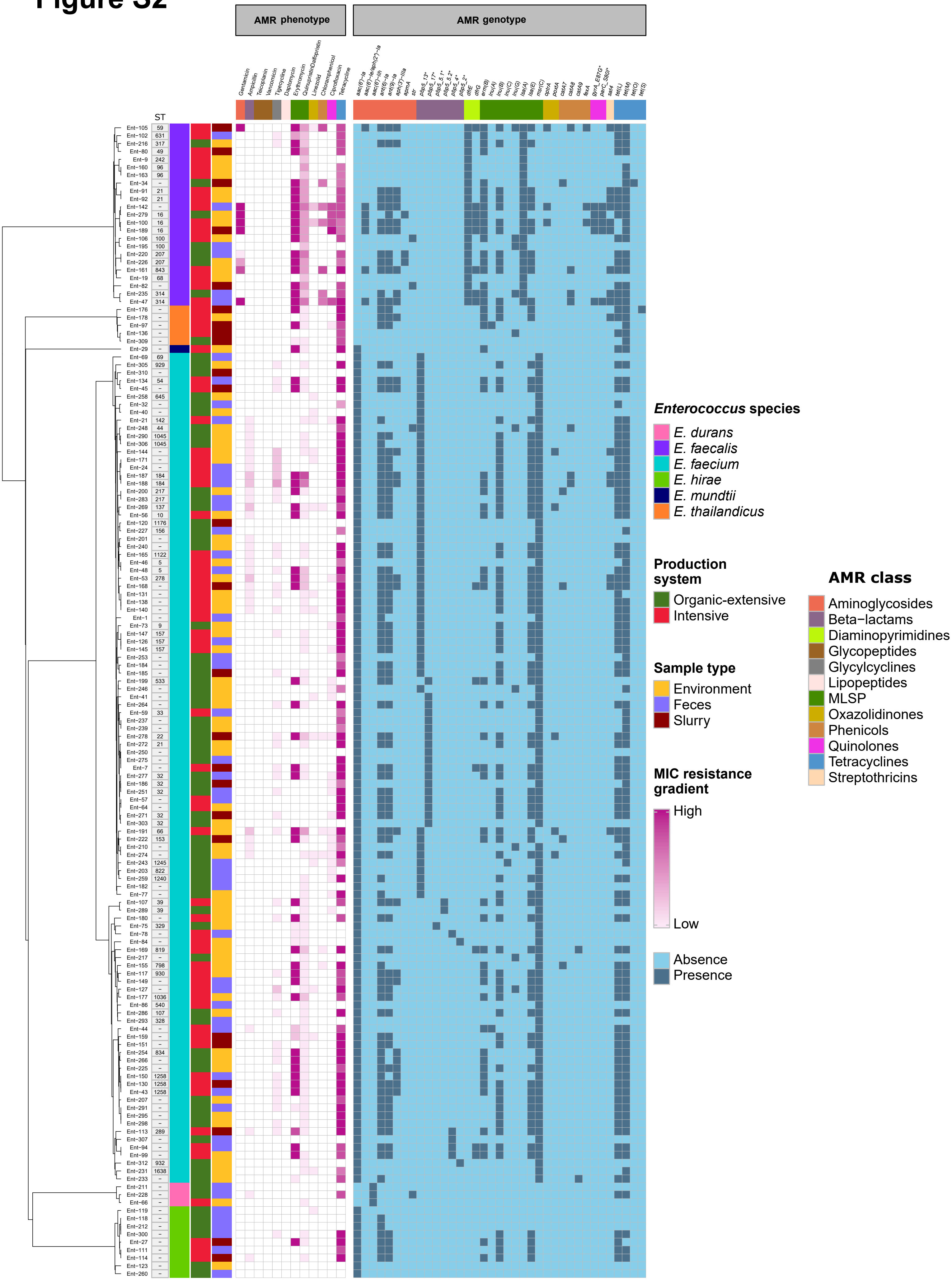
AMR phenotype
AMR genotype
ST
Enterococcus species
E. durans
E. faecalis
E. faecium
E. hirae
E. mundtii
E. thailandicus
Production system
Organic-extensive
Intensive
Sample type
Environment
Feces
Slurry
MIC resistance gradient
High
Low
Absence
Presence
AMR class
Aminoglycosides
Beta−lactams
Diaminopyrimidines
Glycopeptides
Glycylcyclines
Lipopeptides
MLSP
Oxazolidinones
Phenicols
Quinolones
Tetracyclines
Streptothricins

Figure S3

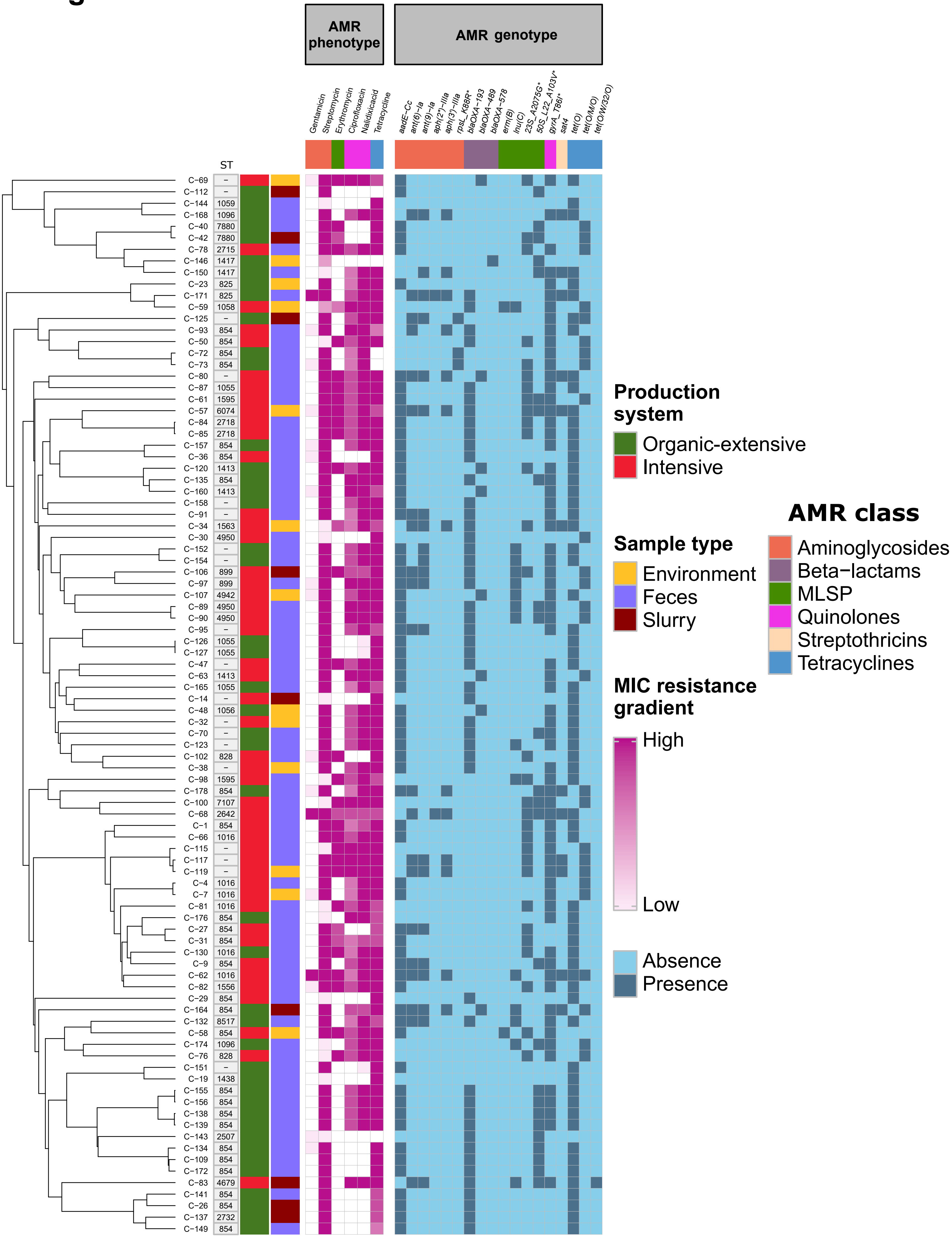

AMR phenotype
AMR genotype
Gentamicin
Streptomycin
Erythromycin
Ciprofloxacin
Nalidixicacid
Tetracycline
aadE−Cc
ant(6)−Ia
ant(9)−Ia
aph(2'')−IIIa
aph(3')−IIIa
rpsL_K88R*
blaOXA−193
blaOXA−489
blaOXA−578
erm(B)
lnu(C)
23S_A2075G*
50S_L22_A103V*
gyrA_T86I*
sat4
tet(O)
tet(O/M/O)
tet(O/W/32/O)
ST
Production system
Organic-extensive
Intensive
Sample type
Environment
Feces
Slurry
MIC resistance gradient
High
Low
Absence
Presence
AMR class
Aminoglycosides
Beta−lactams
MLSP
Quinolones
Streptothricins
Tetracyclines

Figure S4

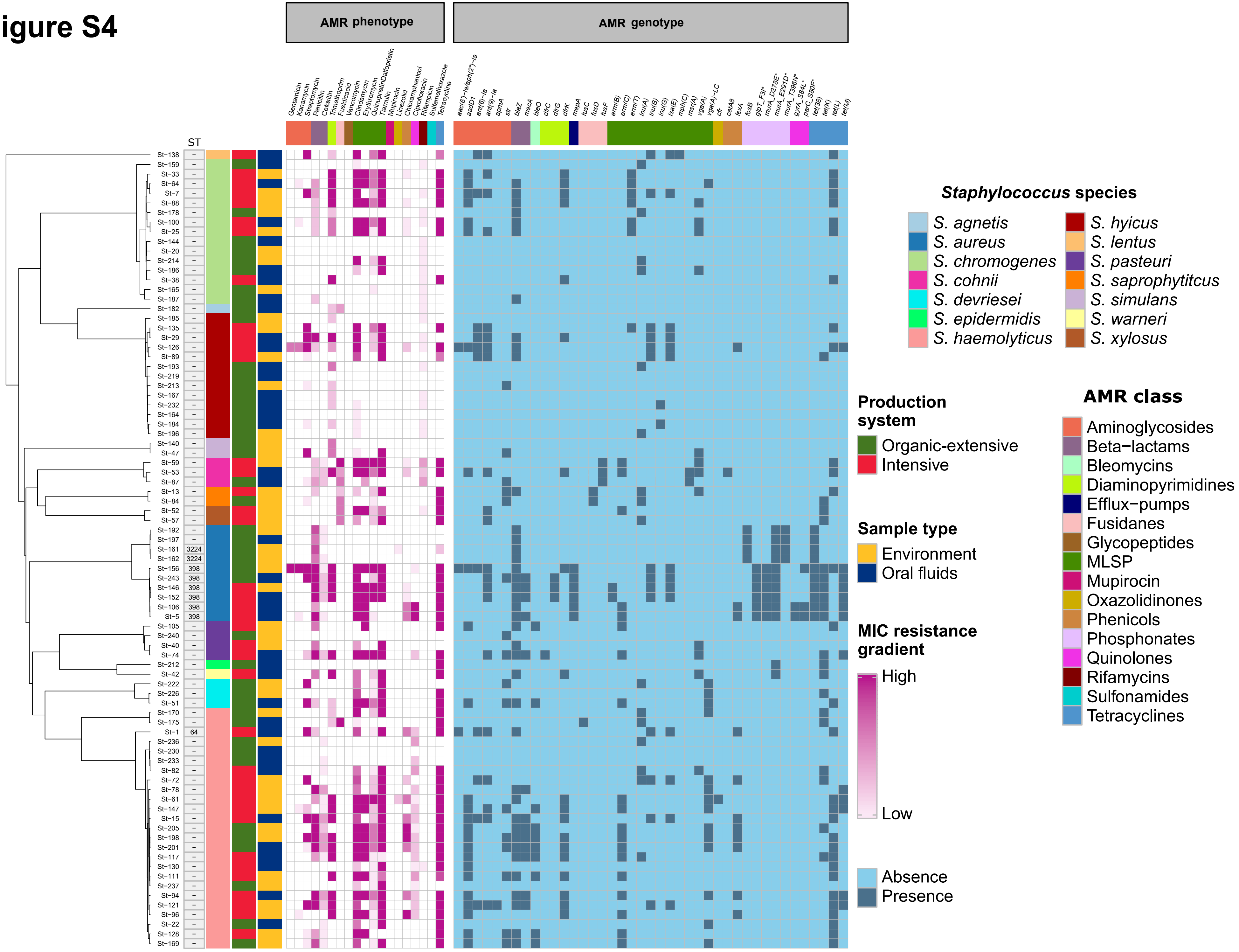
AMR phenotype
AMR genotype
ST
Gentamicin
Kanamycin
Streptomycin
Penicillin
Cefoxitin
Trimethoprim
Fusidicacid
Vancomycin
Clindamycin
Erythromycin
QuinupristinDalfopristin
Tiamulin
Mupirocin
Linezolid
Chloramphenicol
Ciprofloxacin
Rifampicin
Sulfamethoxazole
Tetracycline
Staphylococcus species
S. agnetis
S. aureus
S. chromogenes
S. cohnii
S. devriesei
S. epidermidis
S. haemolyticus
S. hyicus
S. lentus
S. pasteuri
S. saprophytitcus
S. simulans
S. warneri
S. xylosus
Production system
Organic-extensive
Intensive
Sample type
Environment
Oral fluids
MIC resistance gradient
High
Low
Absence
Presence
AMR class
Aminoglycosides
Beta−lactams
Bleomycins
Diaminopyrimidines
Efflux−pumps
Fusidanes
Glycopeptides
MLSP
Mupirocin
Oxazolidinones
Phenicols
Phosphonates
Quinolones
Rifamycins
Sulfonamides
Tetracyclines

Figure S5

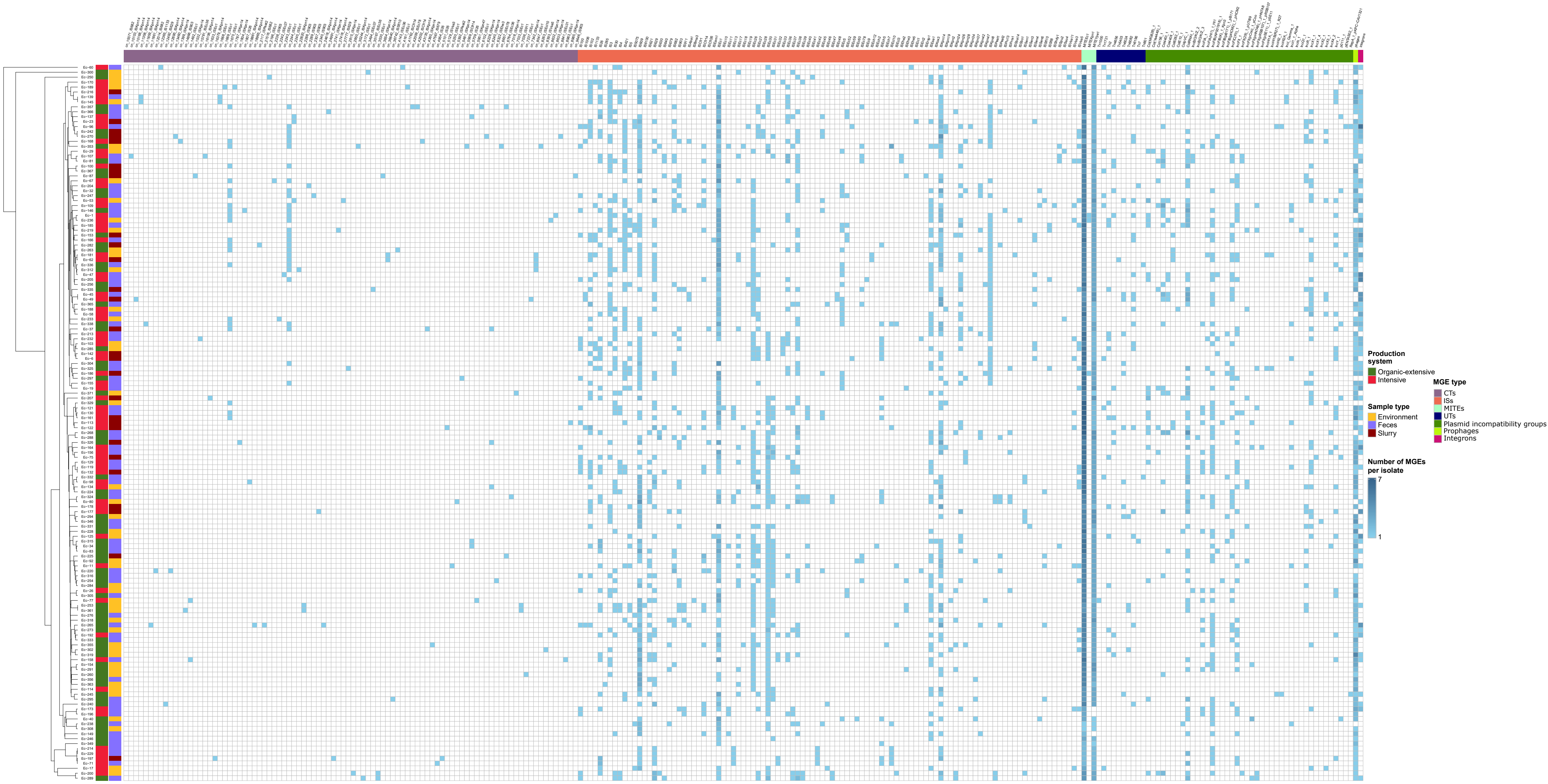

Production system
Organic-extensive
Intensive
Sample type
Environment
Feces
Slurry
Number of MGEs per isolate
7
1
MGE type
CTs
ISs
MITEs
UTs
Plasmid incompatibility groups
Prophages
Integrons

Figure S6

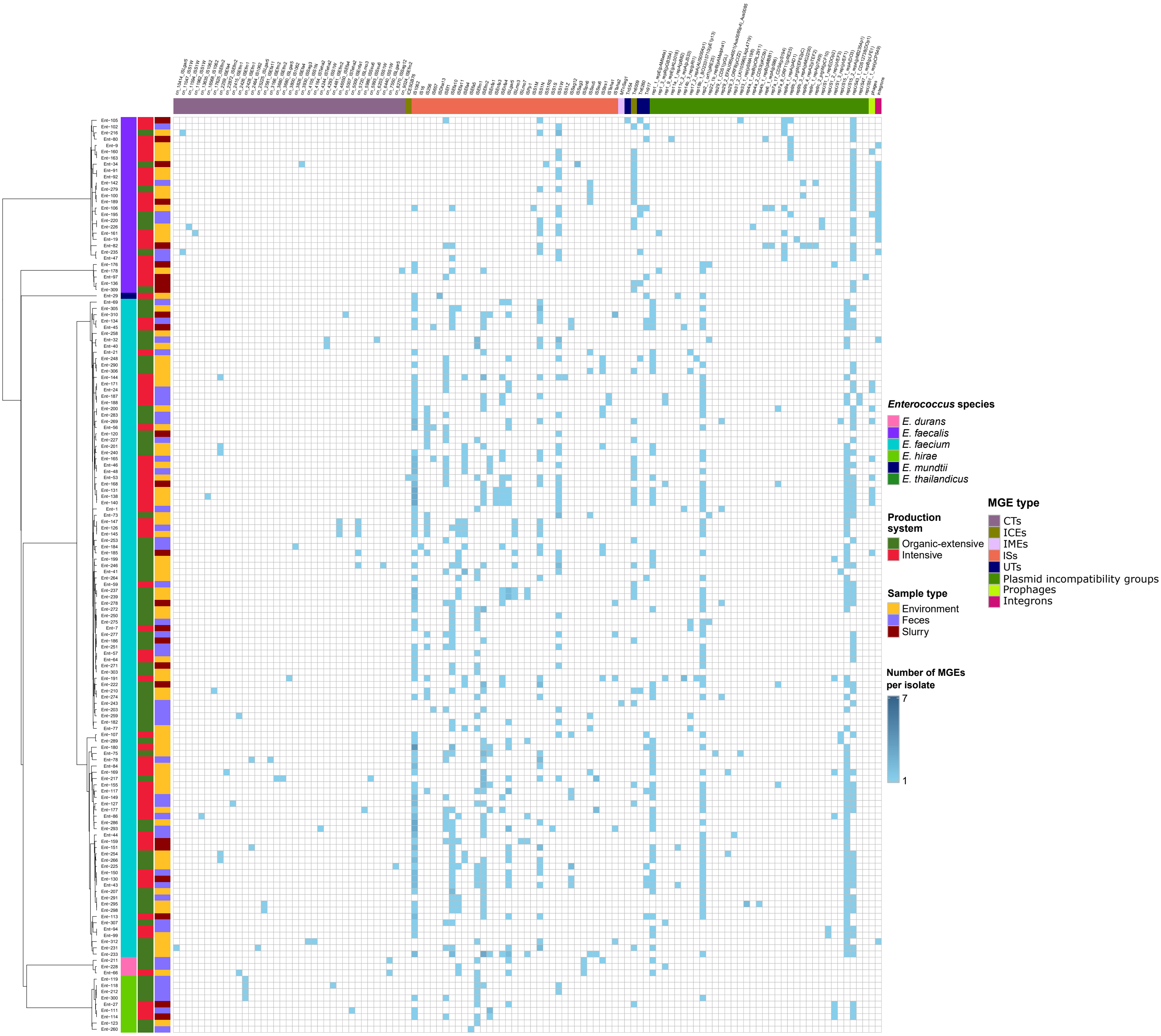
Enterococcus species
E. durans
E. faecalis
E. faecium
E. hirae
E. mundtii
E. thailandicus
Production system
Organic-extensive
Intensive
Sample type
Environment
Feces
Slurry
Number of MGEs per isolate
7
1
MGE type
CTs
ICEs
IMEs
ISs
UTs
Plasmid incompatibility groups
Prophages
Integrons

Figure S7

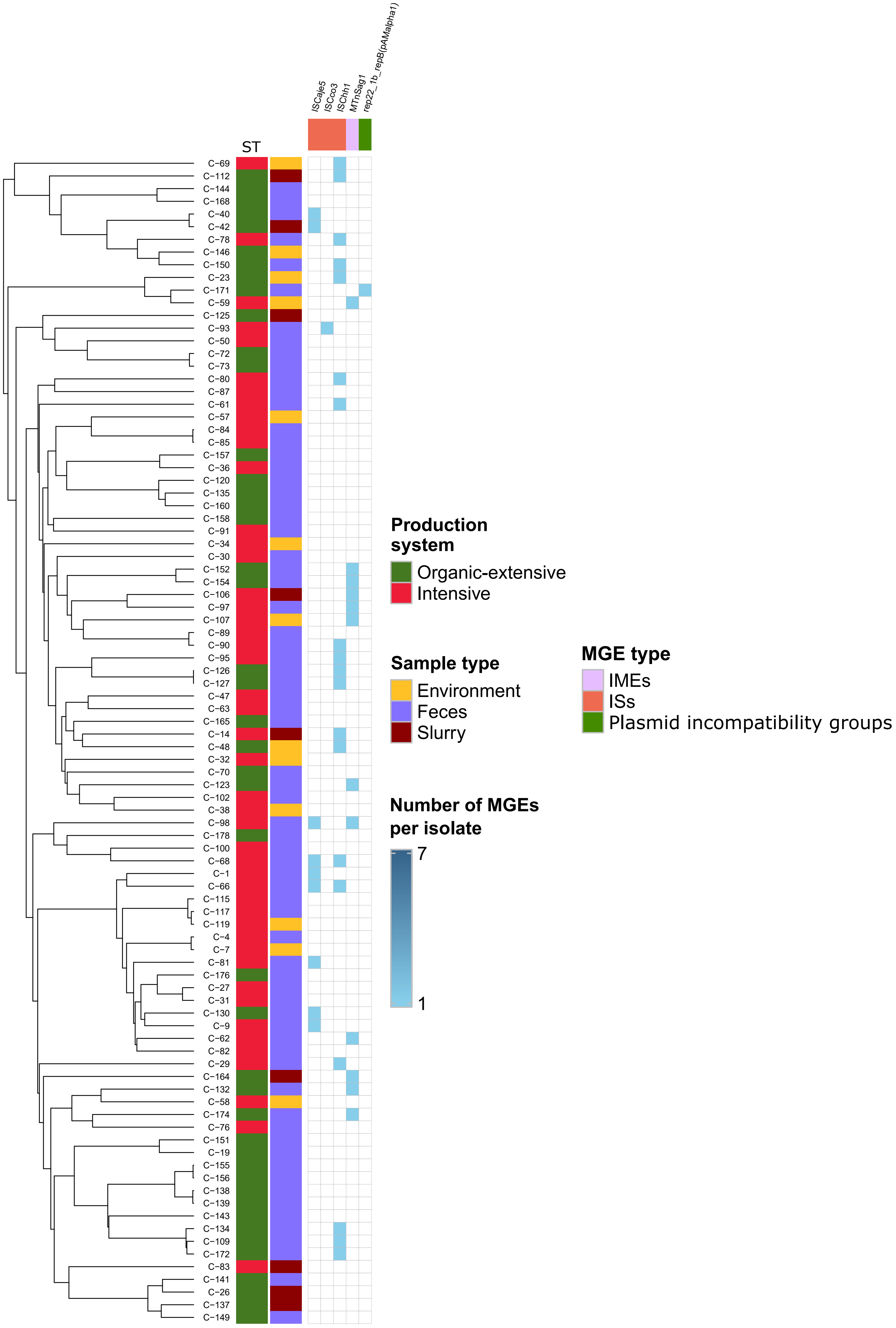

ISCaje5
ISCco3
ISChh1
MTnSag1
rep22_1b_repB(pAMalpha1)
ST
Production system
Organic-extensive
Intensive
Sample type
Environment
Feces
Slurry
MGE type
IMEs
ISs
Plasmid incompatibility groups
Number of MGEs per isolate
7
1

Figure S8

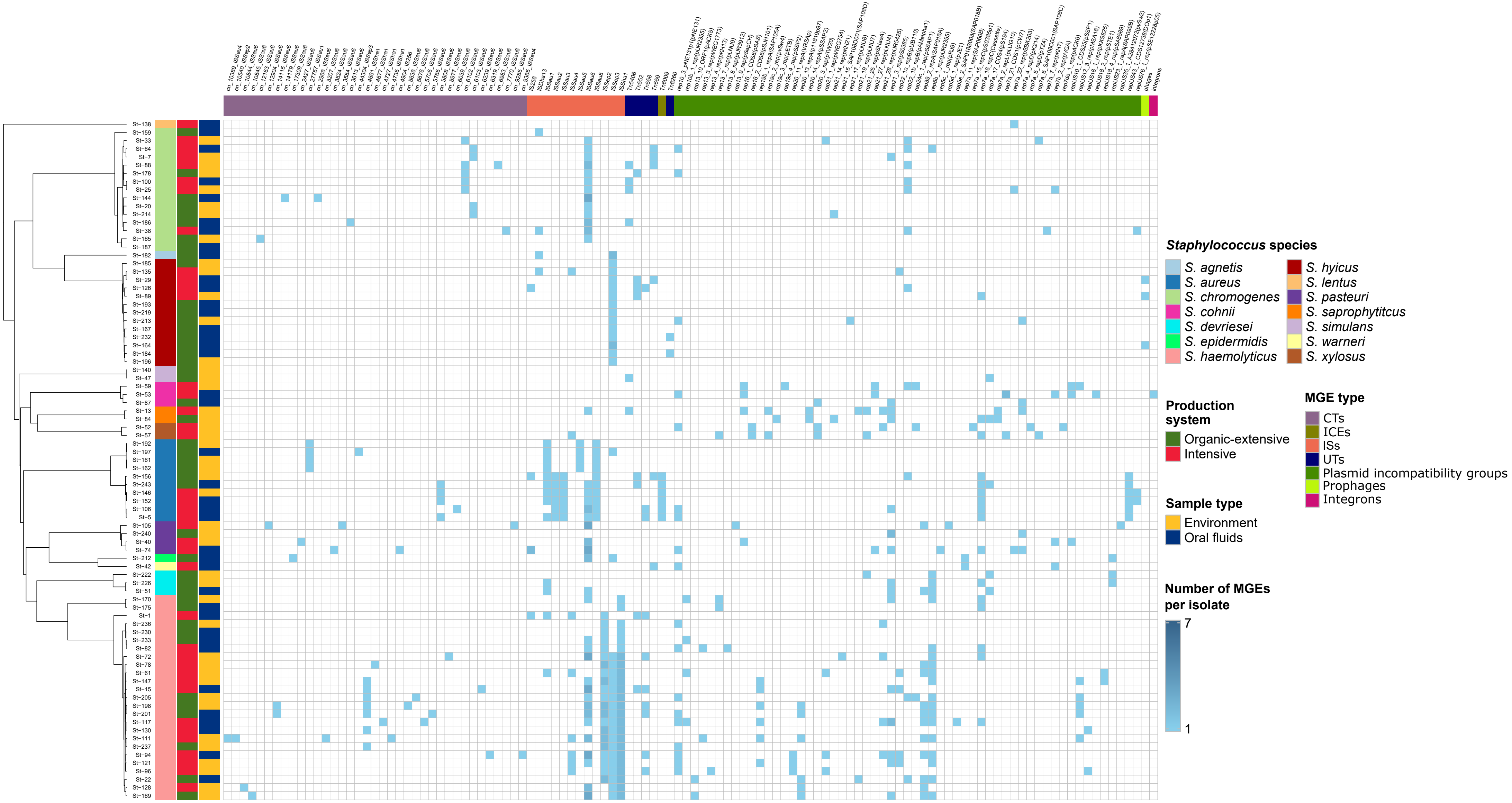

Staphylococcus species
S. agnetis
S. aureus
S. chromogenes
S. cohnii
S. devriesei
S. epidermidis
S. haemolyticus
S. hyicus
S. lentus
S. pasteuri
S. saprophyticus
S. simulans
S. warneri
S. xylosus
Production system
Organic-extensive
Intensive
Sample type
Environment
Oral fluids
MGE type
CTs
ICEs
ISs
UTs
Plasmid incompatibility groups
Prophages
Integrons
Number of MGEs per isolate
7
1

Figure S9

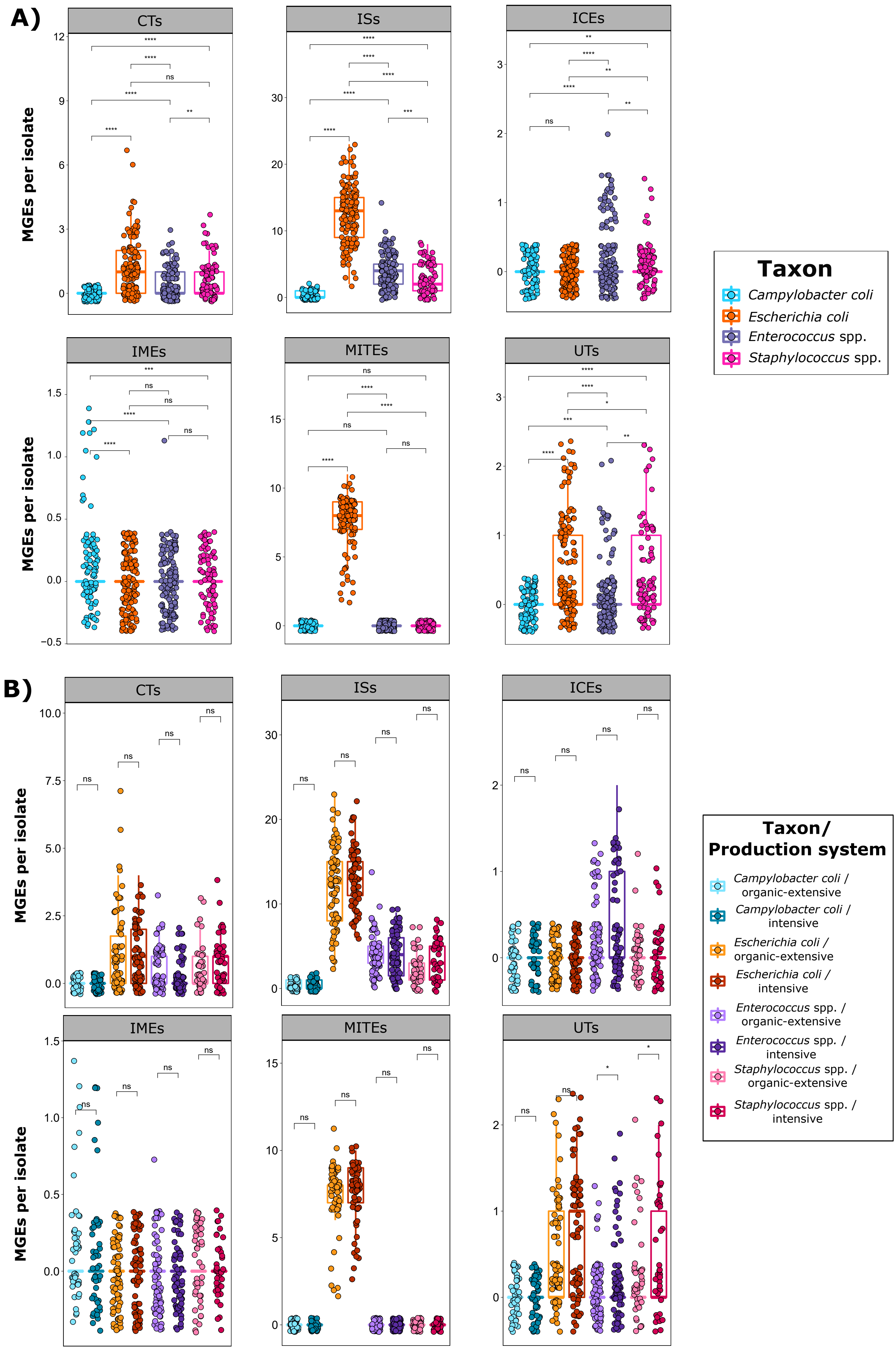

A)
B)
CTs
ISs
ICEs
IMEs
MITEs
UTs
MGEs per isolate
Taxon
Campylobacter coli
Escherichia coli
Enterococcus spp.
Staphylococcus spp.
Taxon/ Production system
Campylobacter coli / organic-extensive
Campylobacter coli / intensive
Escherichia coli / organic-extensive
Escherichia coli / intensive
Enterococcus spp. / organic-extensive
Enterococcus spp. / intensive
Staphylococcus spp. / organic-extensive
Staphylococcus spp. / intensive
